# A scoping review of non-pharmacological perinatal interventions impacting maternal sleep and maternal mental health

**DOI:** 10.1186/s12884-022-04844-3

**Published:** 2022-08-23

**Authors:** Clare Ladyman, Bronwyn Sweeney, Katherine Sharkey, Bei Bei, Tanya Wright, Hannah Mooney, Mark Huthwaite, Chris Cunningham, Ridvan Firestone, T. Leigh Signal

**Affiliations:** 1 Sleep/Wake Research Centre, School of Health Sciences, College of Health, Massey University, PO Box 756, Wellington, 6140 New Zealand; 2 The Warren Alpert Medical School, Brown University, 222 Richmond St, Providence, RI 02903 USA; 3 Turner Institute for Brain and Mental Health, Monash University, 18 Innovation Walk Clayton Campus, Clayton, Victoria 3800 Australia; 4 School of Psychological Medicine, Faculty of Medical and Health Sciences, University of Auckland, Private Bag 92019, Auckland, 1142 New Zealand; 5 Ngāti Raukawa, Te Atiawa, Ngā Rauru, Te Āti Haunui-a-Pāpārangi, School of Social Work, College of Health, Massey University, Private Bag 11-222, Palmerston North, 4442 New Zealand; 6 Department of Psychological Medicine, University of Otago, 23 Mein St, Newtown, Wellington, 6242 New Zealand; 7 Ngāti Raukawa; Ngāti Toarangatira; Te Atiawa; Te Ati Haunui-a-Pāpārangi, Research Centre for Hauora & Health, College of Health, Massey University, PO Box 756, Wellington, 6140 New Zealand; 8 Research Centre for Hauora & Health, College of Health, Massey University, PO Box 756, Wellington, 6140 New Zealand

**Keywords:** Perinatal, Pregnancy, Postnatal, Postpartum, Sleep, Mood, Mental health, Intervention, Health inequities

## Abstract

**Background:**

A woman’s vulnerability to sleep disruption and mood disturbance is heightened during the perinatal period and there is a strong bidirectional relationship between them. Both sleep disruption and mood disturbance can result in significant adverse outcomes for women and their infant. Thus, supporting and improving sleep in the perinatal period is not only an important outcome in and of itself, but also a pathway through which future mental health outcomes may be altered.

**Methods:**

Using scoping review methodology, we investigated the nature, extent and characteristics of intervention studies conducted during the perinatal period (pregnancy to one-year post-birth) that reported on both maternal sleep and maternal mental health. Numerical and descriptive results are presented on the types of studies, settings, sample characteristics, intervention design (including timeframes, facilitation and delivery), sleep and mood measures and findings.

**Results:**

Thirty-seven perinatal interventions were identified and further described according to their primary focus (psychological (*n* = 9), educational (*n* = 15), lifestyle (*n* = 10), chronotherapeutic (*n* = 3)). Most studies were conducted in developed Western countries and published in the last 9 years. The majority of study samples were women with existing sleep or mental health problems, and participants were predominantly well-educated, not socio-economically disadvantaged, in stable relationships, primiparous and of White race/ethnicity. Interventions were generally delivered across a relatively short period of time, in either the second trimester of pregnancy or the early postnatal period and used the Pittsburgh Sleep Quality Index (PSQI) to measure sleep and the Edinburgh Postnatal Depression Scale (EPDS) to measure mood. Retention rates were high (mean 89%) and where reported, interventions were well accepted by women. Cognitive Behavioural Therapies (CBT) and educational interventions were largely delivered by trained personnel in person, whereas other interventions were often self-delivered after initial explanation.

**Conclusions:**

Future perinatal interventions should consider spanning the perinatal period and using a stepped-care model. Women may be better supported by providing access to a range of information, services and treatment specific to their needs and maternal stage. The development of these interventions must involve and consider the needs of women experiencing disadvantage who are predominantly affected by poor sleep health and poor mental health.

## Background

During the perinatal period, physiological changes (including hormonal changes), psychological adjustments, day to day demands of infant and self-care, and managing shifts in employment, finances and relationships all play a part in increasing a woman’s vulnerability to sleep disruption and mood disturbance (e.g., symptoms of depression and anxiety). One in five women will experience significant depressive symptoms during the perinatal period [1] and three in four women will experience insomnia symptoms [2], poor sleep quality [3] or disrupted sleep [4]. The strong bidirectional relationship between sleep and mood is well recognised. Women with greater levels of depressive symptoms report more sleep disruption [5], and women with poor sleep are more likely to experience depressive symptoms both antenatally [6, 7], and postnatally [8, 9].

The sequelae of poor sleep and mood across the perinatal period are significant. Accumulating evidence demonstrates associations between poor sleep and gestational diabetes [10], hypertension [11], pre-term birth [12] and type of birth (i.e., vaginal vs. caesarean) [13]. Studies have also shown that poor mood is associated with adverse obstetric and birth outcomes [14, 15], increased infant mortality and hospitalisation [16], decreases in the likelihood of breastfeeding initiation [17], early breastfeeding cessation [18] and disruptions in parent-baby interactions [19]. The deleterious effects of poor perinatal mental health persist beyond infancy, with children of depressed mothers having impaired or delayed developmental milestones [20] and a fivefold increase of experiencing depression themselves during adolescence [21]. Furthermore, the economic costs of perinatal mental health are significant. For example, a study assessing the costs and health-related quality of life losses over the lifetime of mothers and their children in the United Kingdom found that for all births in 2013, the costs of perinatal anxiety and depression amounted to £6.6 billion, with 60% of the costs relating to the negative impact on children [22].

The perinatal period represents a critical life stage where “there is no health without mental health” [23]. As such, the World Health Organization [24] has highlighted the urgent need for “evidence based, cost effective, and human rights oriented mental health and social care services in community-based settings for early identification and management of maternal mental disorders”. Addressing maternal mental health problems requires many approaches, and whilst pharmacotherapy is the most well studied, it is only indicated for moderate to severe illness. Less than 10% of women consider antidepressants their first choice of treatment [25] and report feeling anxious and regretful and poor adherence when continuing medications during the perinatal period [26, 27]. An international guideline review recommended the use of behavioural interventions before antidepressants as an initial therapy for mild to moderate depression [28].

A broad range of non-pharmacological interventions for improving maternal mental health are increasingly being researched and show promise in their efficacy, acceptability, and accessibility, and improving maternal wellbeing by addressing sleep difficulties is emerging as a viable treatment target. Furthermore, supporting and improving sleep in the perinatal period is not only an important outcome in and of itself, but also a pathway through which future mental health outcomes may be altered. Recent studies have shown that interventions aimed at improving perinatal sleep help reduce depressive symptoms [29, 30] and vice versa; interventions that reduce depressive symptoms assist in improving sleep [31, 32]. Previous reviews have described perinatal interventions that aim to improve sleep or improve mental health [33], or examine the relationship between sleep and mental health during either pregnancy or the postnatal period. To our knowledge, there has been no recently published review of perinatal interventions that influence both maternal sleep and maternal mental health outcomes. Given the bidirectional connections between sleep and mood, the present review is focused on intervention studies that report sleep and mood outcomes concurrently and that are delivered at any point throughout the perinatal period, pregnancy and one year postnatal inclusive.

## Methods

To enable the synthesis of existing knowledge and identify the extent, range, and nature of evidence available on perinatal interventions that have measured the impact on both sleep and mood, a scoping review was conducted. Similar to systematic reviews, scoping reviews use the same systematic, rigorous approach to search databases and extract data [34], but because they are aimed at identifying knowledge gaps, setting research agendas, and/or guiding decision-making, they do not usually include a formal evaluation of the methodological quality of studies [35, 36]. The methodology outlined by Arksey and O’Malley [34], and expanded on by Levac et al. [37] and Tricco et al. [38] (including the PRISMA-ScR reporting guidelines [38]), was used to guide the study processes and reporting. This involved the following: (1) articulation of the research question (2) identification of relevant studies, (3) selection of relevant studies using an iterative team-based approach, (4) charting data, and (5) collating, summarising and reporting results. PROSPERO registration was not possible because it does not accept scoping reviews.

### Identifying the research question

The intent of this review was to summarise current knowledge and identify evidence gaps in this subject area to inform policy and practice and provide recommendations for future research. The research question was defined as: ‘What is the nature, extent and characteristics of interventions developed for perinatal women that influence maternal sleep and maternal mental health?’ This will include identifying when, where and what type of studies have been conducted, describing the study methodologies (settings, sample characteristics, intervention length, facilitation and delivery measures of sleep, mood and acceptability), and summarising sleep and mood findings.

### Inclusion/exclusion criteria

Full-text articles were eligible if published in peer-reviewed academic journals in the English language from 1st January 1975 to 1st April 2021. Randomised controlled trials (RCTs), randomised and non-randomised cross-over studies, and pre-post studies conducted in community, clinical, and mixed settings from any country were included provided they: a) included adequate details of an intervention (duration, methods, analysis techniques); b) the intervention covered any gestational week of pregnancy and/or postnatal week up to 12 months after birth; c) presented sufficient detail of pre- and post-intervention data (either between-group or within- group); d) defined the gestational or postnatal timeframe for intervention and data collection; and e) reported results of at least one circadian OR sleep metric AND at least one measure of mental health. Relevant sleep metrics were based on Buysse’s [39] model of sleep health and could include sleep quantity, quality, latency, continuity, daytime sleepiness and sleep timing. Objective or self-reported measures of sleep were included but measures of fatigue were excluded due to this construct being conceptually and psychometrically distinct from sleepiness [40]. Mental health measures included self-reported and clinician assessments of persistent poor mental or emotional health (such as symptoms of depression and anxiety) but excluded measures of stress (i.e., perceived level of stress, exposure to specific stressors). Pharmacological interventions were excluded as were reviews, meta-analyses, observational studies, case studies, protocols, editorials and conference abstracts. All studies were included irrespective of the sociodemographic or health profile of participants. When study criteria were not clear, authors of the original study were contacted.

### Identifying the relevant studies

The search strategy was co-designed in collaboration with a medical librarian using key search terms for sleep, mood and perinatal, including synonyms and medical subject headings (MeSH terms), and was conducted in the Cochrane Library, PubMed, Medline, psycINFO, Web of Science, CINAHL Complete, and Scopus databases. These databases were systematically searched using the Boolean string: “expect* mother*” OR maternal OR pregnan* OR postnatal OR perinatal OR postpartum OR antepartum OR antenatal AND sleep* OR circadian AND mental OR psycholog* OR psychiatric OR emotional AND intervention* OR treat* OR therap* OR pilot OR feasibility. Non-peer reviewed literature and manual searches of academic manuscript reference lists were also conducted to ensure that all possible interventions matching the inclusion criteria were identified.

### Study selection

**Fig. 1:**
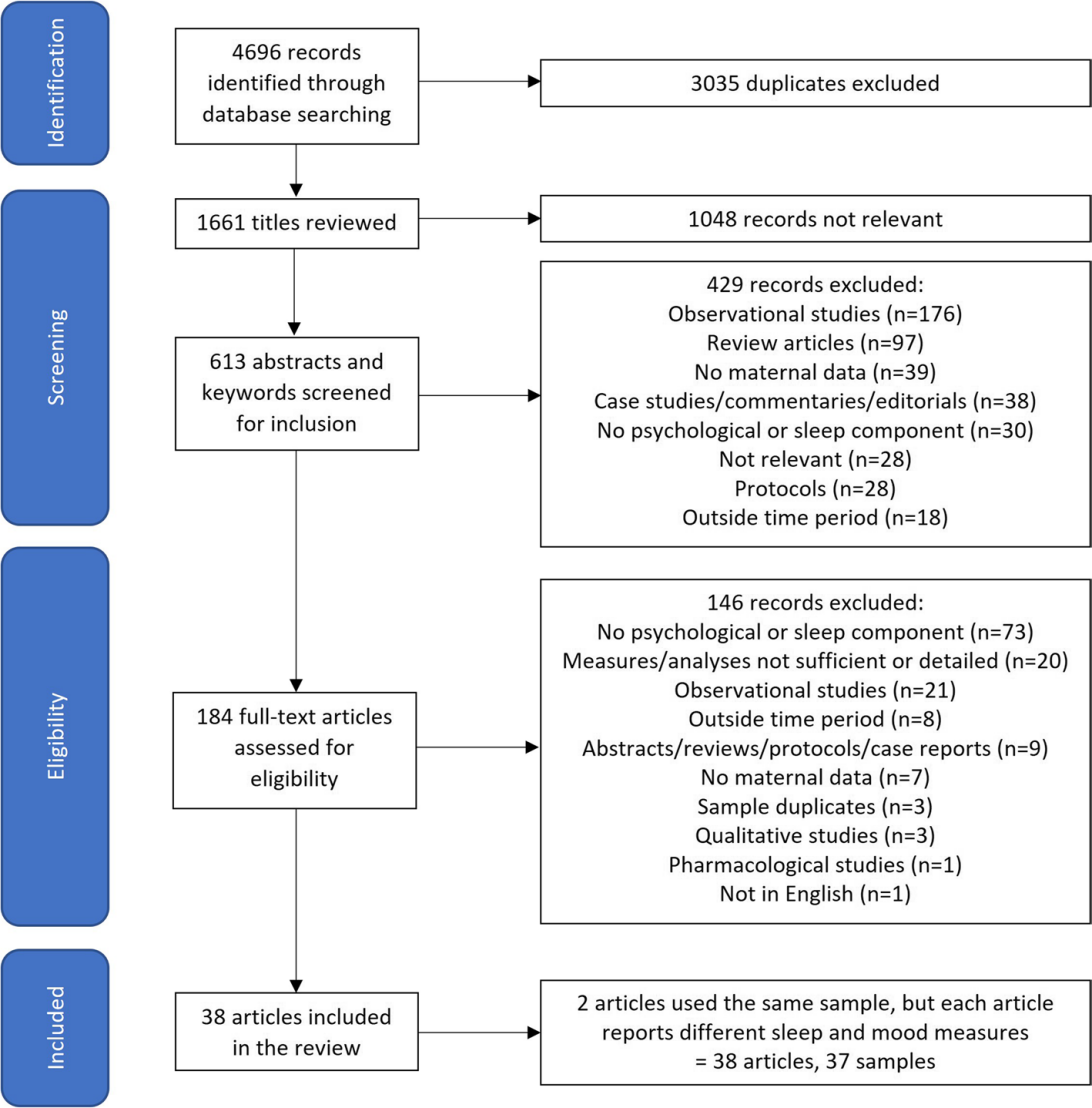
Flow diagram of the study selection process

Endnote reference management software was used to import and manage references. The initial search produced 4696 articles, of which 3035 were duplicates, leaving 1661 for initial screening. Titles, abstracts and keywords of the 1661 identified articles were reviewed and 1477 records were excluded because studies had no intervention component; were not conducted in the perinatal period; the article was a review, case study, protocol or editorial; the study did not include mothers in the intervention; or had no sleep and psychological data (CL). Full text records of the remaining 184 articles were read independently and a further 146 articles were excluded based on the study inclusion/exclusion criteria (CL and TLS). If there was uncertainty about the inclusion of a study, at least two members of the extended research team discussed the study and reached consensus (BS, BB, KS). This resulted in 38 studies considered central to the topic and included in the review. It should be noted that one study reported sleep outcomes for only approximately one third of their sample [41]. Two additional articles used the same sample, but each manuscript reported different sleep and mood measures. Therefore, 38 articles are listed in Table 1 but only 37 study samples/interventions are described. Unless specifically referring to the 38 articles, the remainder of the review refers to the 37 studies/study samples/interventions. Fig. 1 outlines the study selection process.

**Table 1:** Study and sample characteristics

Author, Year, Country	Intervention Focus	Gestational (GE)/ Postnatal (PN) Recruitment Age (mean)	Maternal Age, years (Mean, SD)	Parity (% primiparous)	Education Level	Relationship (% married/ partnered)	Socioeconomic Position	Race/Ethnicity	Physical, Mental and Sleep Health Eligibility Criteria - Healthy - Unknown/No restrictions - Unhealthy
**Avalos et al. (2020), USA **[32]	Mindfulness	0–6 months PN (12 weeks)	30.9 ± 5.2	48%	56% less than college 44% college/university	70%	22% receiving Medicaid	30% Non-Hispanic White 19% Non-Hispanic Black 1% Asian 33% Hispanic 15% Multiracial	Mental Health (PHQ-9 = 10 to 19) Physical Health Sleep Health
**Chang et al. (2015), Taiwan **[42]	Chamomile Tea	6 weeks PN	Intervention 33.2 Control 32.7 (SD not reported)	56%	8% high school 92% college/university	Not reported	51% high 40% middle 9% low	Not reported	Mental Health Physical Health Sleep Health (PSQS ≥16)
**Chen et al. (2015), Taiwan **[43]	Lavender Tea	6 weeks PN	Intervention 32.1 ± 4.0 Control 32.7 ± 3.9	61%	9% high school 91% college/university	Not reported	51% high 40% middle 9% low	Not reported	Mental Health Physical Health Sleep Health (PSQS ≥16)
**Field et al. (1999), USA **[44]	Massage	14–30 weeks GE (23 weeks)	29.5 ± 2.7	Not reported	Not reported	Not reported	27% high 50% upper middle 23% middle	46% White 38% African-American 12% Hispanic 4% Other	Mental Health Physical Health Sleep Health
**Field et al. (2013), USA **[45]	Tai chi/Yoga	13–40 weeks GE (22 weeks)	26.6 ± 5.5	Not reported	Not clearly described	65%	Not clearly described	60% Hispanic 38% African-American 2% Non-Hispanic White	Mental Health (Depression on SCID-I for DSM-IV) Physical Health Sleep Health
**Forsell et al. (2017), Sweden **[46]	CBT	12–28 weeks GE (16 weeks intervention, 19 weeks control)	Intervention 31.2 ± 3.7 Control 30.8 ± 5.3	48%	26% high school 74% college/university	98%	Not reported	Not reported	Mental Health (MDD on DSM-IV and MADRS-S = 15–35 (but no/low suicide risk or other psychiatric disorder) Physical Health Sleep Health
**Galland et al. (2017), New Zealand **[47]	Infant sleep education	28–30 weeks GE	32.0 (SD not reported)	48%	8% did not finish high school 16% high school 76% college/university	Not reported	35% low deprivation 44% medium deprivation 21% high deprivation	85% White 8% Māori/Pacifica 4% Asian 2% Other 1% Middle Eastern/Latin American/ African	Mental Health Physical Health Sleep Health
**Hiscock et al. (2002), Australia **[41]	Infant sleep education	6–12 months PN (8.9 months intervention, 8.6 months control)	Intervention 34.1 ± 3.6 Control 33.3 ± 5.6	Not reported	66% college/university	77%	Not reported	Not reported	Mental Health Physical Health Sleep Health
**Hiscock et al. (2007), Australia **[48]	Infant sleep education	7–12 months PN (7.3 months intervention, 7.4 months control)	Intervention 32.8 ± 4.3 Control 33.2 ± 4.8	47%	17% did not finish high school 32% high school 51% college/university	97%	18% low deprivation 44% medium deprivation 21% high deprivation	80% Australian/New Zealand born (race/ethnicity not reported)	Mental Health Physical Health Sleep Health
**Kempler et al. (2020), Australia **[49]	Infant and maternal sleep education	28–40 weeks GE (mean not reported)	Intervention 33.3 ± 4.0 Control 33.3 ± 4.0	100%	9% high school or diploma 91% college/university	Not reported	Not reported	67% Australian/New Zealand born 33% Other (not specified (race/ethnicity not reported)	Mental Health (no history of major depression) Physical Health Sleep Health
**Kubo et al. (2021), USA **[50]	Mindfulness	9–30 weeks GE (17 weeks)	31.0 yrs. (SD not reported)	70%	11% high school or less 22% some college 67% college graduated	77%	20% Medicaid recipient	59% White 15% Hispanic 11% African-American 4% Asian 11% Other	Mental Health (PHQ-9 = 10 to 19) Physical Health Sleep Health
**Ladyman et al. (2020) New Zealand **[29]	Maternal sleep education	0–14 weeks GE (11.5 weeks)	Intervention 31.5 ± 5.2 Control 31.4 ± 5.1	100%	7% high school 93% college/university	Not reported	Not reported	100% White	Mental Health (previous history but not current) Physical Health Sleep Health (no diagnosed sleep disorder)
**Lee et al. (2013), USA **[51]	Bright Light Therapy	5–10 days PN (mean gestational age of infants when born was 28 weeks)	26.6 ± 6.4	Not reported	Not reported	57%	Not reported	73% Black 13% White 10% Hispanic	Mental Health (no affective illness) Physical Health Sleep Health (no diagnosed sleep disorder, shift work or sleep medications)
**Lewis et al. (2014), USA **[52]	Physical activity	0–8 weeks PN (6 weeks)	31.5 ± 5.0	24%	69% college/university graduated	82%	Not reported	82% White 7% African-American 11% Other	Mental Health (previous history but not current) Physical Health (no hypertension, diabetes, musculoskeletal problems, asthma or any other condition making exercise unsafe) Sleep Health
**Liu et al (2016), Taiwan **[53]	Music therapy	18–34 weeks GE (mean not reported)	Not reported	55%	10% less than senior high school 90% more than senior high school	Not reported	65% high class 28% middle class 7% low class	Not reported	Mental Health Physical Health Sleep Health (PSQI > 5)
**Liu et al. (2021), Taiwan **[54]	Physical activity	6 weeks PN	Intervention 32.2 ± 3.4 Control 33.0 ± 3.3	58%	14% high school 64% college/university 23% graduate	Not reported	52% high 38% middle 10% low	Not reported	Mental Health Physical Health Sleep Health (PSQS ≥16)
**Liu et al. (2021), China **[31]	CBT	Newly delivered, (week not specified)	Intervention 26.9 ± 4.1 Control 27.3 ± 4.6	Not reported	19% junior high school or less 61% senior high school or polytechnic 20% college or above	Not reported	Not reported	Not Reported	Mental Health (EPDS ≥9 and < 13, but no history) Physical Health (no serious underlying disease or severe postpartum complications) Sleep Health
**Manber et al. (2019), USA **[30]	CBT	18–32 weeks GE (25 weeks)	Intervention 33.4 ± 5.2 Control 32.6 ± 4.9	57%	Not reported	Not reported	Not reported	48% White 3% African American 15% Asian 28% Other 6% Unknown	Mental Health (no major depressive, bipolar, panic, posttraumatic stress disorder or thought disorders) Physical Health Sleep Health (DSM-V criteria for insomnia but no other comorbid sleep disorders and not taking medications or having treatment)
**Mendelson et al. (2018), USA **[55]	Mindfulness	Newly delivered (NICU) at 24–39 weeks gestation (mean 31 weeks gestation)	31.0 ± 5.4	Not reported	25% high school 17% some college 8% college associate 17% college graduate 33% graduate school	78%	Not reported	42% African-American 54% White 4% Asian/Pacifica	Mental Health Physical Health Sleep Health
**Mindell et al. (2018), USA **[56]	Massage	3–18 months PN (9 months)	30.6 ± 5.2	Not reported	Not reported	Not reported	Not reported	Not Reported	Mental Health Physical Health (no current acute or chronic illness) Sleep Health
**Ozcan et al. (2020), Turkey **[57]	Breastfeeding, personal hygiene, fatigue, sleep, nutrition and Pilates exercises	Newly delivered, (week not specified)	Intervention 25.2 ± 4.0 Control 25.1 ± 4.5	100%	16% primary school 32% high school 52% college/university	Not reported	Not reported	Not reported	Mental Health Physical Health Sleep Health
**Parry et al. (2019), USA **[58]a	Sleep Restriction	≤34 weeks GE & 0–52 weeks PN (mean not reported)	Not reported	Not reported	Not reported	Not reported	Not reported	Not reported	Mental Health (no bipolar or primary anxiety disorders) Physical Health (no significant medical illness or medication) Sleep Health
**Rouzafzoon et al. (2021), Iran **[59]	Infant sleep education	2–4 months PN (3.2 months intervention, 2.8 months control)	Intervention 30.2 ± 4.6 Control 29.1 ± 2.2	52%	26% primary or high school 74% college/university	Not reported	28% high 67% middle 4% low	Not reported	Mental Health (no diagnosis of depression Physical Health (no uncontrolled chronic disease Sleep Health (no diagnosed sleep disorder)
**Skouteris et al. (2016), Australia **[60]	Health coaching	0–18 weeks GE (16 weeks)	Intervention 31.4 ± 4.9 Control 31.6 ± 4.5	43%	11% high school 89% college/university	92%	Not reported	69% Australian/New Zealander 18% Asian 10% European 2% American 1% African/middle Eastern	Mental Health Physical Health Sleep Health
**Smart et al. (2007), Australia **[61]	Infant sleep education	2–30 weeks PN (15 weeks)	32.6 ± 4.3	65%	92% high school 48% college/university	97%	Not reported	77% Australian/New Zealander (race/ethnicity not reported)	Mental Health Physical Health Sleep Health
**Stremler et al. (2006), Canada **[62]	Infant and maternal sleep education	Newly delivered (week not specified	Intervention 31.1 ± 3.5 Control 32.6 ± 3.5	100%	7% secondary/high school 93% college/university	100%	Not reported	77% White 17% Asian 3% Hispanic 3% Other	Mental Health Physical Health (no poorly controlled chronic illness) Sleep Health (no diagnosed sleep disorders or sleep medications)
**Stremler et al. (2013), Canada **[63]	Infant and maternal sleep education	Newly delivered (mean 1 day postnatal)	Intervention 32.6 ± 5.0 Control 31.8 ± 4.9	100%	2% primary school 7% high school 91% college/university	97%	Not reported	63% White 20% Asian 7% Black 5% Mixed 3% Hispanic 2% Other	Mental Health Physical Health (no poorly controlled chronic illness or postpartum complications) Sleep Health (no diagnosed sleep disorders or sleep medications)
**Sun et al. (2021), China **[64]	Mindfulness	12–20 weeks GE (14 weeks)	29.9 ± 4.0	65%	15.4 yrs. year of schooling (mean)	100%	Not reported	99% Han 1% Hui	Mental Health (EPDS > 9 or PHQ-9 > 4 but no/low suicide risk or other psychiatric disorder and no psychiatric medication or treatment) Physical Health Sleep Health
**Swanson et al. (2013), USA **[65]	CBTi	0–12 months PN (6 months)	30.0 ± 7.0	Not reported	42% some college 42% college graduate 17% postgraduate	83%	Not reported	67% White 17% African-American 8% Native American 8% Multiracial	Mental Health (MINI = MDD and EPDS> 11 but no other psychiatric disorders) Physical Health Sleep Health (ISI > 7, daytime impairment, TWT-*N*= > 60 min 3 nights/week for at least 1 month)
**Swanson et al. (2018), USA **[66]	Bright Light Therapy	0–6 months PN (14 weeks)	32.3 ± 3.3	Not reported	20% some college 80% bachelor’s degree or higher	90%	Not reported	80% White 20% African-American	Mental Health (DSM-V = MDD and SIGH-SAD ≥20 but no other psychiatric disorders) Physical Health Sleep Health (ISI > 7)
**Teychenne et al. (2020), Australia **[67]	Physical activity	3–9 months PN (mean not reported)	Intervention 33.6 ± 3.7 Control 33.0 ± 3.7	Not reported	26% no tertiary education 74% tertiary education	97%	Not reported	Not reported	Mental Health (EPDS ≥10, but not taking antidepressants) Physical Health Sleep Health
**Tomfohr-Madsen et al. (2017), Canada **[68]	CBTi	12–28 weeks GE (19 weeks)	31.0 ± 3.7	62%	69% bachelor degree or higher	100%	Not reported	77% White	Mental Health (no bipolar or psychotic disorders and no antidepressant or hypnotic medications) Physical Health Sleep Health (ISI > 12, but no OSA or RLS)
**Wilson et al. (2019), Australia **[69]	Infant sleep education	3–23 months PN (9 months)	34.5 ± 4.2	54%	88% tertiary degree or higher	90%	Not reported	Not reported	Mental Health Physical Health Sleep Health
**Wilson et al. (2019), Australia **[70]	Infant sleep education	3–23 months PN (9 months)	*Note: Same sample as above						Mental Health Physical Health Sleep Health
**Xue et al. (2020), China **[71]	Magnolia tea	Newly delivered (week not specified	25.4 ± 4.6	80%	28% high school or less 56% college/university 16% graduate school	95%	Not reported	Not Reported	Mental Health Physical Health Sleep Health (PSQS ≥16)
**Yang et al. (2018), Taiwan **[72]	Physical activity	6 weeks PN	31.9 yrs. (SD not reported)	67%	10% high school 71% college/university 19% graduate school	Not reported	50% high 43% middle 7% low	Not Reported	Mental Health Physical Health Sleep Health
**Zhao et al. (2017), China **[73]	Maternal mental health education	0–28 weeks GE (21 weeks intervention, 22 weeks control)	Intervention 30.4 ± 3.6 Control 30.6 ± 3.9	100%	2% elementary school or lower 8% middle school 31% vocational college 59% college/university	Not reported	Not reported	Not Reported	Mental Health (EPDS ≥9 or PDSS ≥60 but not MDD) Physical Health (obstetric complication) Sleep Health
**Zhao et al. (2020), China **[74]	Maternal mental health education	28–35 weeks GE (30 weeks)	Intervention 30.7 ± 3.4 Control 30.0 ± 3.2	100%	10% middle school or lower 22% vocational college 51% college/university degree 17% masters degree or above	100%	Not reported	Not Reported	Mental Health (EPDS ≥9 but not MDD) Physical Health Sleep Health

*DSM-IV* Diagnostic and Statistical Manual of Mental Disorders, fourth edition, *DSM-V* Diagnostic and Statistical Manual of Mental Disorders, fifth edition, *EPDS* Edinburgh Postnatal Depression Scale, *ISI* Insomnia Severity Index, *MADRS-S* Montgomery-Åsberg Depression Rating Scale - self report, *MINI* Mini-International Neuropsychiatric Interview, *MDD* Major Depressive Disorder, *OSA* Obstructive Sleep Apnea, *PDSS* Postpartum Depression Screen Scale, *PHQ-9* 9-item Patient Health Questionnaire, *PSQI* 19-item Pittsburgh Sleep Quality Inventory, *PSQS* 14-item Postpartum Sleep Quality Scale, *RLS* Restless Leg Syndrome, *SIGH-SAD* Structured Interview Guide for the Hamilton Depression Rating, *TWT-N* Total Wake Time Nocturnal

### Charting the data

**Fig. 2:**
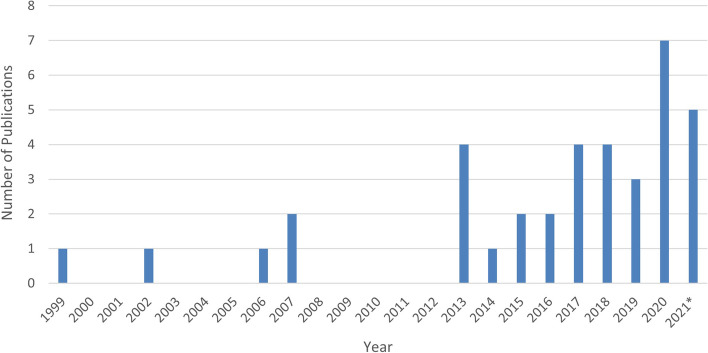
Publications per year

**Fig. 3:**
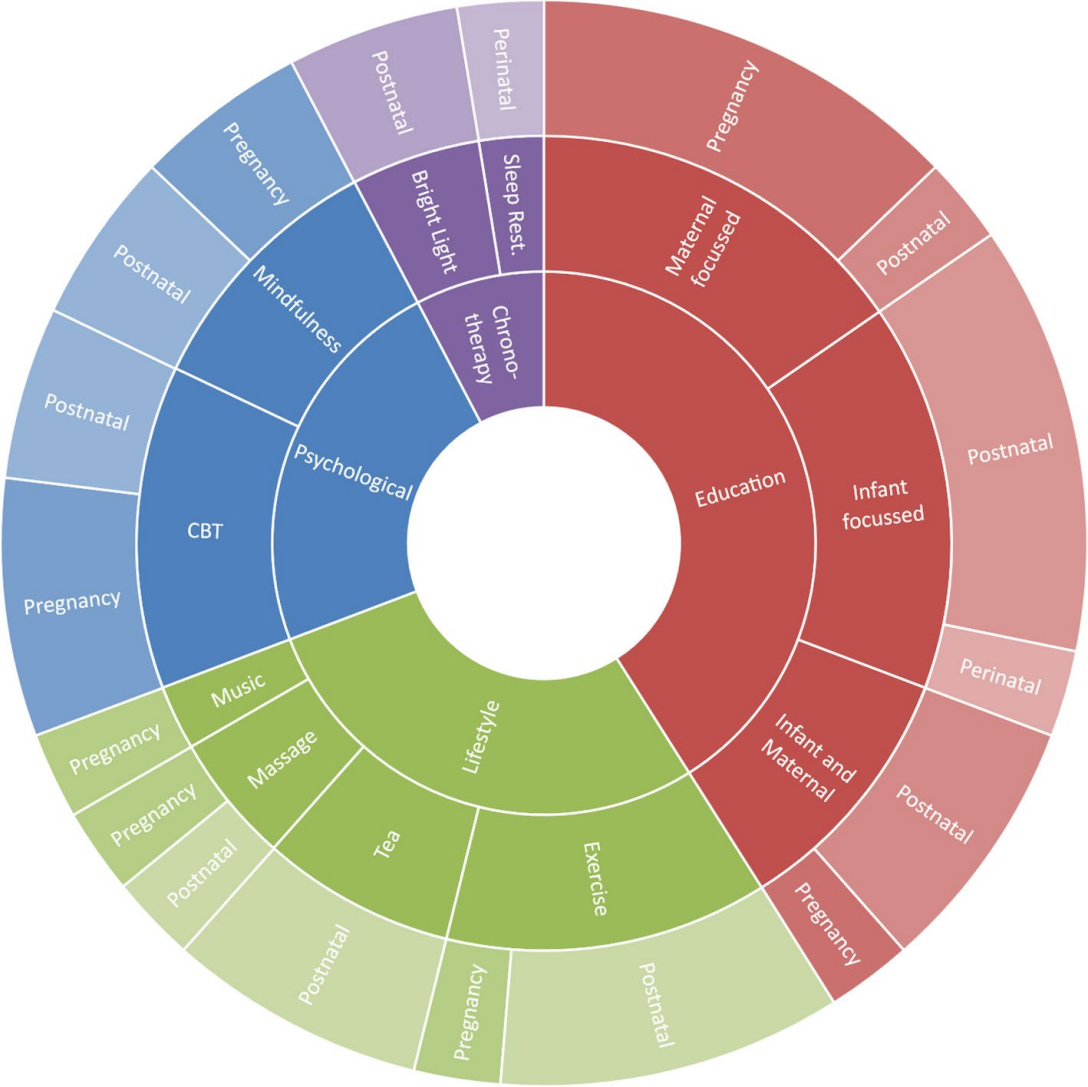
Intervention categories and time periods

**Fig. 4:**
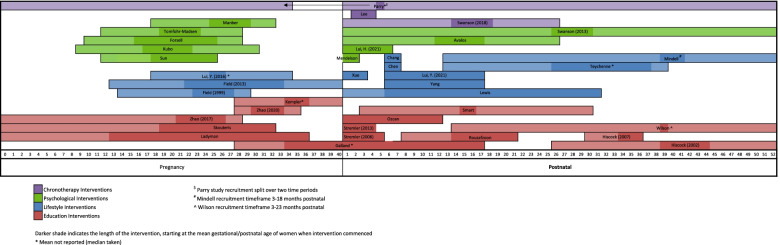
Timelines of perinatal intervention studies including recruitment and intervention phases

A data extraction form was created jointly by CL and TLS in Excel and included the study characteristics and information outlined in Tables 1, 2, 3, 4, 5, 6, 7 and Figs. 2, 3, and 4. Figures 2, 3 and 4 illustrate the number of publications per year, intervention categories/timeframes and a timeline of intervention studies respectively. Table 1 describes the author(s), year of publication, country in which the research was conducted, intervention focus and participant demographics, including gestational/postnatal age at enrolment, maternal age, parity, education level, socioeconomic position, race/ethnicity and whether participants were required to meet physical, mental or sleep-related criteria. Tables 2 and 3 summarise the sample characteristics and the sleep and mental health measures respectively. Details on each study are summarised in Tables 4, 5, 6 and 7 (depending on the type of intervention) and include study design, method of intervention delivery, facilitator, length, data collection time points, number of participants (including breakdown of enrolled/completed, case/control and retention rates), recruitment sites/methods, and the sleep and mood variable(s) assessed. A summary of sleep and mood results are also included in these tables as a secondary outcome of the review.

**Table 2:** Sample characteristics

	Number of studies reporting demographic variable (proportion of participants with this data^a^)	Proportion in a category ^b^	range, median
**Parity**	26 (81%)	68% primiparous (7 studies were 100% primiparous)	Range 24–100% Median 61%
**Education Level**	30 (85%)	74% with at least some tertiary/ college/university education	range 20–100%, median 76%
**Relationship Status**	21 (43%)	91% married/partnered	range 65–100%, median 92%
**Socioeconomic Position**	11 (37%)	84% considered high or middle class (or low to medium deprivation)	range 78–100% median 91%
**Race/Ethnicity**	18 (44%)	65% white	range 0–100% median 63%

^a^ Proportion of participants with data on this variable as a percentage of the total number of participants in the review

^b^ Proportions based only on studies that reported data

**Table 3:** Sleep and mental health measures used

Sleep Measure	Studies Using Measure	Psychological Measure	Studies Using Measure
PSQI	18	EPDS	30
Actigraphy	7	STAI	5
ISI	6	PHQ	4
PSQS	6	GAD	4
Sleep diary	5	SIGH-SAD	3
ESS	4	DASS	3
GSDS	4	CES-D	2
Sleep quality analogue scale ^a,b,c,d^	4	HAM A/D	2
Sleep quantity analogue scale ^e,f,g,h^	4	SF-12/SF-36	2
Subjective sleep duration (hrs)	4	POMS-D	1
VSH	2	PAAS	1
Self-reported sleep problem	2	MADRS-S	1
DLMO	2	DSM-IV (SCID-I)	1
PAD	1	BMIS	1
CIRENS	1	WHOQOL-PH	1
Subjective sleep latency (mins)	1	QIDS-SR	1
GSQ	1	PSA	1
No. of good nights sleep (per week)^i^	1	IDA-I	1
PSG	1	PDSS	1
KSS	1	**Total**	65
**Total**	75		

*BMIS* Brief Mood Introspection Scale, *CES-D* Centre for Epidemiological Studies-Depression, *CIRENS* Circadian Energy Scale, *DASS* Depression, Anxiety, and Stress Scale, *DLMO* Dim light melatonin onset, *DSM-IV (SCID-I)* Structured Clinical Interview for DSM-IV Axis I Disorders, *EPDS* Edinburgh Postnatal Depression Scale, *ESS* Epworth Sleepiness Scale, *GAD* Generalized Anxiety Disorder Scale, *GSDS* General Sleep Disturbance Scale, *GSQ* Generalized Sleep Questionnaire, *HAM A/D* Hamilton Anxiety/Depression Rating Scale, *IDA-I* Irritability Depression Anxiety (Irritability), *ISI* Insomnia Severity Index, *KSS* Karolinska Sleepiness Scale, *MASRS-S* Montgomery-Åsberg Depression Rating Scale (self-report), *PAAS* Perinatal Anxieties and Attitudes Scale, *PAD* Phase angle difference between DLMO and midpoint of sleep per wrist actigraphy, *PDSS* Postpartum Depression Screening Scale, *PHQ* Patient Health Questionnaire, *POMS-D* Profile of Mood States (depression), *PSA* Pregnancy-Specific Anxiety Measure, *PSG* Polysomnography, *PSQI* Pittsburgh Sleep Quality Inventory, *PSQS* Postpartum Sleep Quality Scale, *QIDS-SR* Quick Inventory of Depressive Symptoms (self-report), *SF-12/SF-36* Medical Outcomes Survey Short Form (12 or 36 item), *SIGH-SAD* Structured Interview Guide for the Hamilton Depression Rating Scale, Seasonal Affective Disorders, *STAI* State Anxiety Inventory, *VSH-15* Verran and Snyder-Halpern Sleep Scale, *WHOQOL-PH* World Health Organisation Quality of Life Psychological Health Subscale

^a^ 8-point scale from 1 = “very bad” to 8 = “very good”

^b^ 9 cm visual scale from “very good” to “very bad”

^c^ 4-point scale and dichotomised at the midpoint into “good” vs “bad”

^d^ 4-point scale from “not nearly good enough” to “more than good enough”

^e^ 8-point scale from 1 = “not nearly enough” to 8 = “more than enough”

^f^ 9 cm visual scale from “more than enough” to “not nearly enough”

^g^ 4-point scale and dichotomised at the midpoint into “enough” vs “not enough”

^h^ 4-point scale from “not nearly enough” to “more than enough”

^i ^“In the last week, how often did you get a good night’s sleep?” dichotomized into good (> 3 nights/week) or poor (≤3 nights/week)

**Table 4:** Psychological interventions

Author	Intervention Description	Study Design / Analytical Approach	Intervention Delivery	Intervention Facilitator	Intervention Length	Data Time Points (PN = postnatal, GE = gestational)	Number of Participants (Intervention vs Control)	Retention Rate	Recruitment Sites and Methods	Mental Health Measures	Sleep Measures	Results Summary
**Avalos et al. (2020), USA **[32]	Mindfulness meditations using Headspace™	Single arm, pre-post	App based, self-delivered after instruction	No personal contact	10–20 min per day for 6 weeks	• Baseline (0–6 months PN) • Intervention end	27 enrolled 19 completed	70%	Obstetrics and gynecology clinics	PHQ-8	PSQI-19	• Improved sleep quality • Reduced depressive symptoms
**Forsell et al. (2017), Sweden **[46]	Self-guided internet delivered CBT program with readings, assessments, worksheets and written feedback and support	RCT, within and between groups	Online, self-guided with regular written feedback	CBT-trained therapist	10 weeks	• Baseline (12–28 weeks GE) • Intervention end	42 enrolled (22 vs 20) 39 completed (21 vs 18)	93%	Social media advertisements and posters/ flyers in maternity clinics	MADRS-S-9 SCID-I EPDS-10 GAD-7	ISI-7	• No change within or between groups for insomnia symptoms • Reduced depressive and anxiety symptoms pre-post (MADRS and GAD) • Fewer depressive symptoms in intervention group post intervention (MADRS and SCID-I) but no difference between groups on the GAD and EPDS
**Kubo et al. (2021), USA **[50]	Mindfulness meditations using Headspace™	Single arm, pre-post	App based, self-delivered after instruction	No personal contact	10–20 min per day for 6 weeks	• Baseline (9–30 weeks GE) • Intervention end	27 enrolled 20 completed	74%	Obstetrics and gynecology clinics	PHQ-8	PSQI-19	• Improved sleep quality • Reduced depressive symptoms
**Liu (H.) et al. (2021), China **[31]	CBT including psychological evaluation, self-activity plan, mental health education, delivery and post-birth care and greater social support	RCT, within and between group	Not reported	Not reported	1 hr. weekly sessions for 6 weeks	• Baseline (newly delivered) • Intervention end	260 Enrolled (130 vs 130) 228 completed (113 vs 115)	88%	Municipal hospital	EPDS-10 HAM-A HAM-D	PSQI-19	• Improved sleep quality pre-post • Intervention group better sleep quality • Reduced depressive and anxiety symptoms pre-post (EPDS & HAM-D/A) • Intervention group fewer depressive and anxiety symptoms scores (EPDS & HAM-D/A) and less women with clinically relevant depression (EPDS)
**Manber et al. (2019), USA **[30]	CBTi plus education on infant sleep development and elements from ‘Tips for Improving Postpartum Sleep’ program	RCT, between group	Face-to-face individual sessions	Trained CBT therapists	Weekly sessions for 5 weeks	• Baseline (18–32 weeks GE) • Intervention end	194 enrolled (96 vs 98) 134 completed (71 vs 63)	69%	University/county hospital–based obstetric clinics and community advertisements	EPDS-9	ISI-7 TWT-A TWT-S	• Intervention groups greater reductions in insomnia severity, faster remission of insomnia disorder and less self-reported nocturnal wake time, but no difference in objective nocturnal wake time • Reduced EPDS scores in both groups
**Mendelson et al. (2018), USA **[55]	Mindfulness intervention including a video introduction and four 5- and 10-min audio mindfulness practices	Single arm, pre-post	Audio recordings, self-delivered after instruction	No personal contact	2 weeks	• Baseline (newly delivered) • Intervention end	27 enrolled 24 completed	89%	Hospital neonatal intensive care unit	PHQ-8 GAD-7	PSQI-SQ PSQI-D PSQI-SL PSQI-SE PSQI-SM	• Improved sleep quality and sleep duration but not sleep efficiency or latency pre-post • Reduced depressive and anxiety symptoms scores
**Sun et al. (2021), China **[64]	Self-guided mindfulness on custom built smartphone app	RCT, between group	App based, self-delivered after instruction	No personal contact	15–25 min per day, 6 days a week for 8 weeks	• Baseline (12–20 weeks GE) • Mid intervention (4 weeks after allocation) • Intervention end (8 weeks after allocation) • Late pregnancy (18 weeks after allocation) • 6 weeks PN	168 enrolled (84 vs 84) 92 completed (52 vs 40)	55%	Hospital obstetric clinic	EPDS-10 GAD-7	PSQI-19	• Intervention group fewer depressive symptoms (EPDS) immediately post intervention and late pregnancy but not mid intervention or at 6 weeks postnatal • Intervention group lower anxiety scores (GAD-7) mid and immediately post intervention and at both follow-up timepoints • Intervention group had fewer depressive and anxiety symptoms compared to control • No difference between groups in sleep quality at any timepoint
**Swanson et al. (2013), USA **[65]	Modified CBTi including stimulus control, sleep restriction, sleep hygiene, relaxation strategies and infant sleep	Single arm, pre-post	Face-to-face individual sessions	Clinical psychologist certified in behavioral sleep medicine	45–60 min weekly session for 5 weeks	• Baseline (0-6 months PN) • Intervention end	16 enrolled 12 completed	75%	Mood Disorders Clinic	EPDS-9 QIDS-SR-16	ISI-7 PSQI-19 TST-D TWT-D SE-D	• Improvements in sleep efficiency, total wake time, total sleep time, insomnia severity (ISI) and sleep quality (PSQI) • Reduced depressive symptoms
**Tomfohr-Madsen et al. (2017), Canada **[68]	CBTi including review of sleep diary, sleep education, stimulus control, cognitive sleep strategies and relapse prevention	Single arm, pre-post	Face-to-face group sessions	Licensed, PhD-level clinical psychologist and a clinical psychology doctoral trainee	90 min weekly sessions for 5 weeks	• Baseline (12–28 weeks GE) • Intervention end	14 enrolled 13 completed	93%	Physicians’ offices, midwifery services and childbirth education classes	EPDS-10 EPDS-9 PSA-40	ISI-7 PSQI-19 TIB-A TST-A SOL-A SE-A WASO-A Awak-A TIB-D TST-D SE-D SOL-D Awak-D	• Improved insomnia symptoms (ISI) and sleep quality (PSQI) • Less actigraphic TIB, shorter SOL and increased SE, and increased sleep diary TST and SE, but no change in actigraphic WASO and TST or sleep diary TIB, SOL and awakenings • Reduced depressive symptoms (EPDS) and pregnancy-specific anxiety symptoms (PSA)

*EPDS-9* 9-item Edinburgh Postnatal Depression Scale (sleep item removed), *GAD-7* 7-item Generalized Anxiety Disorder Scale, *HAM-A* Hamilton Anxiety Rating Scale, *HAM-D* Hamilton Depression Rating Scale, *ISI-7* 7-item Insomnia Severity Index, *MADRS-S-9* 9-item Montgomery-Åsberg Depression Rating Scale - self report, *PHQ-8* 8-item Patient Health Questionnaire (suicidal thoughts item removed), *PSQI-19* 19-item Pittsburgh Sleep Quality Inventory, *PSQI-SQ* Pittsburgh Sleep Quality Inventory Sleep Quality subscale, *PSQI-D* Pittsburgh Sleep Quality Inventory Duration subscale, *PSQI-SL* Pittsburgh Sleep Quality Inventory Sleep Latency subscale, *PSQI-SE* Pittsburgh Sleep Quality Inventory Sleep Efficiency subscale, *PSQI-SM* Pittsburgh Sleep Quality Inventory Sleep Medication Use subscale, *PSA-40* 40-item Pregnancy-Specific Anxiety Measure, *QIDS-SR-16* 16-item Quick Inventory of Depressive Symptoms (self-report), *Awak-A* Awakenings (actigraphy), *Awak-D* Awakenings (sleep diary), *SCID-I* Structured Clinical Interview for DSM Axis I Disorders, *SE-A* Sleep Efficiency (actigraphy), *SE-D* Sleep Efficiency (sleep diary), *SOL-A* Sleep Onset Latency (actigraphy), *SOL-D* Sleep Onset Latency (sleep diary), *TIB-A* Time in Bed (actigraphy), *TIB-D* Time in Bed (sleep diary), *TST-A* Total Sleep Time (actigraphy), *TST-D* Total Sleep Time (sleep diary), *TWT-A* Total Wake Time (actigraphy), *TWT-D* = Total Wake Time (sleep diary), *WASO-A* Wake After Sleep Onset (actigraphy)

**Table 5:** Educational interventions

Author	Intervention Description	Study Design / Analytical Approach	Intervention Delivery	Intervention Facilitator	Intervention Length	Data Time Points (PN = postnatal, GE = gestational)	Number of Participants (Intervention vs Control)	Retention Rate	Recruitment Sites and Methods	Mental Health Measures	Sleep Measures	Results Summary
**Galland et al. (2017), New Zealand **[47]	Infant sleep education with four arms: control; sleep; **f**ood, **a**ctivity and **b**reastfeeding (FAB); combined (sleep and FAB) including group sessions, information booklet, consultant session and home visits for mothers and partners	Four armed RCT, between group	Face-to-face, group sessions and individual sessions at home	Researcher with infant sleep training and international board-certified lactation consultant	2 (sleep), 4 (FAB) or 6 (combined) sessions over a max of 6 months	• Baseline (28–30 weeks GE) • 4 months PN • 6 months PN	802 enrolled (205 FAB vs 192 Sleep vs 196 Combination vs 209 control) 784 completed (201 FAB vs 187 Sleep vs 189 Combination vs 207 control)	98%	Maternity hospital	EPDS-10	Sleep Quality^a^ Sleep Quantity^b^ TST-S SOL-S	• No difference between groups for maternal sleep quantity or quality scores, duration or long sleep latency (≥30 min) • No difference between groups for depressive symptoms
**Hiscock et al. (2002), Australia **[41]	Education on infant sleep and infant sleep problems with a sleep management plan involving controlled crying or ‘camping out’	RCT, between group	Face-to-face individual sessions	Senior paediatric trainee with one year’s sleep management experience	3 sessions fortnightly for 6 weeks	• Baseline (6–12 months PN) • 2 months after randomisation • 4 months after randomisation	166 enrolled (78 vs 78) 164 completed (2 months) (76 vs 76) 156 completed (4 months) (75 vs 71) ^a^*Sleep data is only reported on the last 57 recruited mothers*	99% (2 months) 94% (4 months)	Maternal and Child Health Centres	EPDS-10	Sleep Quality^c^ Sleep Quantity^d^	• Intervention group more likely to have “very good” (sleep quality) and “enough” (sleep duration) at 2 months but no difference at 4 months • Depression scores did not differ between groups at 2 months, but after controlling for professional services, baseline depression score and group allocation the intervention group had greater improvement. No difference at 4 months • Subgroup with initial depression scores EPDS ≥10, greater improvement in the intervention group at 2 months and at 4 months
**Hiscock et al. (2007), Australia **[48]	An individualised sleep management plan involving controlled crying or ‘camping out’, with handouts on infant sleep, infant sleep problems, dummies and overnight feeding	RCT, between group	Face-to-face individual sessions	Maternal and child health nurses trained by paediatrician and child psychologist	One phone consultation and one follow-up appt over 3 weeks	• Baseline (7 months PN) • 10 months PN • 12 months PN	328 enrolled (174 vs 154) 319 completed (168 vs 151)	97%	Invitations from Maternal and Child Health nurses	EPDS-10 SF-12	Sleep Quality^e^ Sleep Quantity^f^	• Intervention group less poor and insufficient sleep at 12-months postnatal but not at 10-months postnatal • Intervention group had fewer depressive symptoms (EDPS) and better mental health score (SF-12) at 10- and 12-months postnatal • Intervention effect on depression symptoms at 10 months postnatal was greater for mothers with initial EPDS score was > 9 but no difference at 12-month postnatal
**Kempler et al. (2020), Australia **[49]	Psychoeducation intervention including a booklet covering science behind sleep, normal sleep changes during pregnancy and postpartum, association between sleep and perinatal depression, infant sleep and settling routines, and strategies.	RCT, between group	Face-to-face group sessions	Sleep psychologist	Two 1.5-hour sessions for 2 weeks	• Baseline (third trimester) • Intervention end (6 weeks PN) • 4 months PN • 10 months PN	215 enrolled (107 vs 108) 178 completed (89 vs 89)	83%	Prenatal classes at large hospital, social media and word of mouth	EPDS-10 DASS-D DASS-A	PSQI-19 ISI-7 ESS-8 GSQ	• Intervention group better sleep quality and fewer insomnia symptoms at 4 months, but no difference at 6 weeks or 10 months postnatal • No difference between groups for daytime sleepiness • Control group almost twice as likely to score > 10 on the PSQI (no difference on scores > 5) • Control group 4 times more likely to meet criteria for clinical insomnia (ISI > 15) • Intervention group more likely to nap • No difference between groups for depression or anxiety
**Ladyman et al. (2020) New Zealand **[29]	Trimester specific sleep education sessions and booklet covering general sleep and circadian information, how and why sleep changes in each trimester; and trimester-specific sleep support strategies.	Single arm, with an additional between group comparison	Face-to-face individual sessions	Sleep scientist	Three 45–90 min sessions over 24 weeks	• Baseline (0–14 weeks GE) • Intervention end (36 weeks GE) • 12 weeks PN	15 intervention 76 matched controls	68% (end of pregnancy) 64% (12 week follow up)	Online and traditional media and community advertisements	EPDS-10 EPDS-D EPDS-A	TST-24 GNS GSDS-21-T GSDS-SQ GSDS-MI GSDS-OI ESS-8	• Intervention group better sleep quality and sleep continuity immediately post intervention, but no difference at 12-week postnatal • Intervention group better sleep latency immediately post intervention and at follow-up • No difference between groups for sleep duration and daytime sleepiness • Intervention group fewer depressive symptoms (total EPDS and depression subscale) immediately post intervention, but no difference 12-weeks postnatal • No difference between groups for anxiety symptoms
**Ozcan et al. (2020), Turkey **[57]	Nursing care program containing information about breastfeeding, personal hygiene, fatigue, sleep, nutrition and Pilates exercises.	RCT, within and between group	Face-to-face individual sessions	Registered nurse	Eight 60–120 min sessions over 12 weeks	• Baseline (newly delivered) • Intervention end	117 enrolled (58 vs 59) 110 completed (55 vs 55)	94%	Hospital delivery room	WHOQOL-PH	PSQI-19 PSQI-SQ PSQI-SL PSQI-D PSQI-SE PSQI-SDis PSQI-SM PSQI-DD	• Improvements in pre-post sleep latency, duration, sleep disturbances and daytime disfunction (PSQI total score and respective subscales) (no difference in sleep quality and sleep efficiency) • Intervention group better sleep quality, sleep latency, sleep disturbances and daytime disfunction (PSQI total score and respective subscales) (no difference in sleep duration and sleep efficiency) • Intervention group mood remained stable while control group deteriorated
**Rouzafzoon et al. (2021), Iran **[59]	Preventive behavioural sleep intervention (BSI) including infant sleep education and infant sleep strategies	RCT, between group	Face-to-face individual sessions and follow-up phone calls	Researcher/ midwife (lead author)	One 90 min session with weekly phone calls for 8 weeks	• Baseline (2–4 months PN) • Intervention end	92 enrolled (41 vs 41) 83 completed (37 vs 36)	90%	Health centres	EPDS-10	PSQI-19	• Improved intervention group sleep quality • Intervention group fewer depressive symptoms
**Skouteris et al. (2016), Australia **[60]	Promotion of healthy lifestyle behaviours and mood management and body image issues that during pregnancy	RCT, between group	Face-to-face group and individual sessions	Trained health coach (an allied health professional)	One 1 hr. individual and one 2 hr. group sessions with weekly phone calls for 5 weeks	• Baseline (0–18 weeks GE) • 33 weeks GE (1 week post intervention)	261 enrolled (130 vs 131) 172 completed (T2) (84 vs 96) 172 completed (T3) (75 vs 85)	66% at T2 65% at T3	Large antenatal clinic or small satellite clinic	EPDS-10 DASS-A	PSQI-19	• Intervention group had better sleep quality post intervention • No difference between groups for depressive or anxiety symptoms
**Smart et al. (2007), Australia **[61]	Consultation for mothers and partners on infant safety, infants’ behavioural sleep problems plus written management plan and follow up consultation	Single arm, pre-post	Face-to-face individual sessions	Paediatrician or trainee paediatrician	One 1 hr. session with follow-up appointment 2 weeks later	• Baseline (2 weeks-7 months PN) • 3 weeks (1 week after intervention end)	72 enrolled 59 completed	82%	Paediatric outpatient clinic	EPDS-10	Sleep Quantity^g^ Sleep Quality^h^	• Improved sleep quality • No improvement for sleep quantity • Improved depressive symptoms and the number of women with clinically significant scores reduced
**Stremler et al. (2006), Canada **[62]	Behavioural sleep educational intervention including education on maternal sleep hygiene and sleep support, infant sleep structure, issues and strategies	RCT, between group	Face-to-face individual session and follow-up phone calls	Study nurse	One 45-60 min session with 5 weekly phone calls for 5 weeks	• Baseline (newly delivered) • Intervention end	30 enrolled (15 vs 15) 30 completed (15 vs 15)	100%	Hospital postpartum unit	EPDS-10 EPDS-10- > 12 STAI-20-T STAI-20-Mod	GSDS-T-21 GSDS-PS TST-NA TST-DA TST-24-A Awak-A WASO-A LNSP-A SRSP	• Intervention group longer sleep durations • Fewer women in the intervention group rated their sleep as a problem • No difference between groups for sleep quality (GSDS), 24 hour or daytime TST, longest nocturnal sleep period, WASO or awakenings • No difference between groups for depression (EPDS) or anxiety (STAI) scores or clinically significant depression or anxiety scores
**Stremler et al. (2013), Canada **[63]	Behavioural sleep educational intervention including education on maternal sleep hygiene and sleep support, infant sleep structure, issues and strategies	RCT, between group	Face-to-face individual session and follow-up phone calls	Sleep intervention nurse	One 45-60 min session with 3 weekly phone calls for 4 weeks	• Baseline (newly delivered) • Intervention end • 12 weeks PN	246 enrolled (123 vs 123) 215 completed (110 vs 105)	87%	Hospital postpartum unit	EPDS-10	GSDS-T TST-NA Awak-A	• No differences between groups for sleep duration, quality (GSDS) or awakenings • No differences between groups for depressive symptoms
**Wilson et al. (2019), Australia **[69] ^a^***Note: Same sample as below***	Multidisciplinary intervention offering maternal and infant sleep opportunities, psychoeducation, medical and psychological support, and supervised practice implementing infant settling strategies	Single arm, pre-post	Residential program	Midwives, lactation consultants, nurses, psychologists, general practitioners, paediatricians, psychiatrists	5 days residential program	• Baseline (2–23 months PN) • Intervention end	85 enrolled 78 completed	92%	Early parenting centre	EPDS-10	TST-D SE-D Awak-D WASO-D SOL-D TSTNap-D PSQI-19 CIRENS-2 ESS-8 KSS-1	• Improved sleep efficiency, awakenings, WASO, SOL, sleep quality, daytime sleepiness and KSS but not sleep duration) • Improved depressive symptoms
**Wilson et al. (2019), Australia **[70] ^a^***Note: Same sample as above***	Multidisciplinary residential intervention offering maternal and infant sleep opportunities, psychoeducation, medical and psychological support, and supervised practice implementing infant settling strategies.	Single arm, pre-post	Residential program	Midwives, lactation consultants, nurses, psychologists, general practitioners, paediatricians, psychiatrists	5 days	• Baseline (2–23 months PN) • Intervention end	85 enrolled 78 completed	92%	Early parenting centre	DASS-D DASS-A DASS-S IDA-I-6	ISI-7 TST-D	• Improved insomnia symptoms (ISI) but not sleep duration • Improved depressive and anxiety symptoms (DASS subscales) and irritability symptoms (IDA-I)
**Zhao et al. (2017), China **[73]	Couple-separated psychoeducational workshops for first-time parents. Five sessions for mothers on maternal mental health and the last session specific for husbands	RCT, between group	Face-to-face group sessions	Midwives (with psychological research experience)	Six 1.5 hr. sessions for 6–12 weeks	• Baseline (0–28 weeks GE) • 42 days PN	352 enrolled (176 vs 176) 334 completed (167 vs 167)	95%	Obstetrics and gynecology hospital antenatal clinic	EPDS PDSS	TST-S	• Intervention group longer sleep durations • Intervention group less minor (EPDS 9–12 or PDSS 60–79) and major (EPDS ≥13 or PDSS ≥80) depression
**Zhao et al. (2020), China **[74]	Psychoeducational workshops for first-time parents focused on perinatal mental health and breastfeeding	RCT, between group	Face-to-face group sessions	Midwives (certified lactation consultant with psychological research experience)	Four 60 min sessions for 4–8 weeks	• Baseline (28–35 weeks GE) • 3 days PN	182 enrolled (91 vs 91) 180 completed (91 vs 89)	99%	Maternal hospital antenatal clinic	EPDS-10	TST-S	• Intervention group longer sleep durations • Intervention group fewer depressive symptoms

*CIRENS-2* 2-item Circadian Energy Scale, *DASS-D* Depression, Anxiety, and Stress Scale - Depression Subscale, *DASS-A* Depression, Anxiety, and Stress Scale - Anxiety Subscale, *DASS-S* Depression Anxiety Stress Scale -Stress Subscale, *EPDS-10* 10-item Edinburgh Postnatal Depression Scale, *EPDS-10- > 12* 10-item Edinburgh Postnatal Depression Scale Score > 12, *EPDS-D* Edinburgh Postnatal Depression Scale Depression subscale, *EPDS-A* Edinburgh Postnatal Depression Scale Anxiety subscale, *ESS-8* 8-item Epworth Sleepiness Scale, *GNS* Good Night’s Sleep (dichotomized into ‘good’ (> 3 nights/wk) or ‘poor’ (≤3 nights/wk)), *GSDS-T-21* 21-item General Sleep Disturbance Scale Total, *GSDS-SQ* General Sleep Disturbance Scale Sleep Quality subscale, *GSDS-MI* General Sleep Disturbance Scale Maintenance Insomnia subscale, *GSDS-OI* General Sleep Disturbance Scale Onset Insomnia subscale, *GSDS-PS* General Sleep Disturbance Scale Poor Sleeper (Score > 42), *GSQ* Generalized Sleep Questionnaire, *IDA-I-6* 6-item Irritability Depression Anxiety Irritability Subscale, *ISI-7* 7-item Insomnia Severity Index, *KSS-1* 1-item Karolinska Sleepiness Scale, *PDSS* Postpartum Depression Screen Scale, *PSQI-19* 19-item Pittsburgh Sleep Quality Inventory, *PSQI-SQ* Pittsburgh Sleep Quality Inventory Sleep Quality subscale, *PSQI-D* Pittsburgh Sleep Quality Inventory Duration subscale, *PSQI-SL* Pittsburgh Sleep Quality Inventory Sleep Latency subscale, *PSQI-SE* Pittsburgh Sleep Quality Inventory Sleep Efficiency subscale, *PSQI-SM* Pittsburgh Sleep Quality Inventory Sleep Medication Use subscale, *PSQI-SDis* Pittsburgh Sleep Quality Inventory Sleep Disturbance subscale, *PSQI-SM* Pittsburgh Sleep Quality Inventory Sleep Medication subscale, *PSQI-DD* Pittsburgh Sleep Quality Inventory Daytime Dysfunction subscale, *SRSP* Self-reported Sleep Problem, *SF-12* 12-item Short Form Health Survey, *STAI-20-T* 20-item State-Trait Anxiety Inventory Total score, *STAI-20-Mod* State-Trait Anxiety Inventory Score Moderate (score between 40 and 59), *WHOQOL-PH* World Health Organisation Quality of Life Assessment Scale psychological health subscale, *Awak-D* Awakenings (diary), *Awak-A* Awakenings (actigraphy), *LDSP* Longest Daytime Sleep Period (actigraphy), *LNSP-A* Longest Nocturnal Sleep Period (actigraphy), *SE-D* Sleep Efficiency (diary), *SOL-D* Sleep Onset Latency (diary), *SOL-S* Sleep Onset Latency (actigraphy), *TST-NA* Total Sleep Time (Nocturnal −9:00 PM-9:00 AM) (actigraphy), *TST-DA* Total Sleep Time Daytime (9:00 AM-9:00 PM) (actigraphy), *TST-24-A* Total Sleep Time in 24 hrs (actigraphy), *TST-D* Total Sleep Time (diary), *TST-S* Total Sleep Time (self-report), *TSTNap-D* Total Sleep Time Nap (diary), *TST-24-S* Total Sleep Time in 24 hrs (self-report), *WASO-A* Wake After Sleep Onset (actigraphy), *WASO-D* Wake After Sleep Onset (diary)

^a^ Sleep Quality analogue scale (8-point scale from 1 = “very bad” to 8 = “very good”)

^b^ Sleep Quantity analogue scale (8-point scale from 1 = “not nearly enough” to 8 = “more than enough”)

^c^ Sleep Quality analogue scale (9cm visual scale from “very good” to “very bad”)

^d^ Sleep Quantity analogue scale (9cm visual scale“more than enough” vs “not nearly enough”

^e^ Sleep Quality analogue scale (4-point ordinal scale, dichotomised at the midpoint into “good” vs “bad”)

^f^ Sleep Quantity analogue scale (4-point ordinal scale, dichotomised into "enough" vs "not enough"

^g^ Sleep Quantity analogue scale (4-point scale, 1 =“not nearly enough”, to 4 =“more than enough”

^h^ Sleep Quality analogue scale (4-point scale, 1 = “not nearly good enough”, to 4 =”more than good enough”

**Table 6:** Lifestyle interventions

Author	Intervention Description	Study Design / Analytical Approach	Intervention Delivery	Intervention Facilitator	Intervention Length	Data Time Points (PN = postnatal, GE = gestational)	Number of Participants (Intervention vs Control)	Retention Rate	Recruitment Sites and Methods	Mental Health Measures	Sleep Measures	Results Summary
**Chang et al. (2015), Taiwan **[42]	Drinking chamomile tea	RCT, between group	Self-delivered after instruction	No personal contact	One cup per day for 2 weeks	• Baseline (6 weeks PN) • Intervention end • 10 weeks PN	80 enrolled (40 vs 40) 73 completed (35 vs 37)	91%	Teaching hospital	EPDS-10	PSQS-F1 PSQS-F2	• Intervention group better sleep efficiency related to physical-symptoms immediately post intervention but not at 4 weeks (PSQS-F1) • No difference between groups in sleep efficiency related to infant night-care and daytime dysfunction (PSQS-F2) • Intervention group fewer depressive symptoms immediately post intervention and at 4 weeks
**Chen et al. (2015), Taiwan **[43]	Drinking lavender tea	RCT, between group	Self-delivered after instruction	No personal contact	One cup per day for 2 weeks	• Baseline (6 weeks PN) • Intervention end • 10 weeks PN	80 enrolled (40 vs 40) 76 completed (38 vs 38)	95%	Medical centre clinic	EPDS-10	PSQS-14	• No difference between groups for sleep quality • Intervention group fewer depressive symptoms immediately post intervention but not at 4 weeks, (difference also seen at baseline)
**Field et al. (1999), USA **[44]	Full body massage therapy with mother in side-lying position	RCT, between and within group	Face-to-face, individual sessions	Trained massage therapists	Ten 20-min massages twice weekly for 5 weeks	• Baseline (14–30 weeks GE) • First day of intervention (before/after treatment) • Last day of intervention (before/after treatment) • Intervention end	26 enrolled (14 vs 12) 26 completed (14 vs 12)	100%	Obstetrics and gynecology clinics and community advertisements	STAI-20 POMS-D CES-D-20 PAAS-P PAAS-L PAAS-W PAAS-PO	VSH-D VSH-E VSH-S	• Within group improvements in immediate (beginning to end of massage) depressive (POMS) and anxiety (STAI) symptoms • Intervention (within group) reported less disrupted sleep post intervention (VHS-D) • No difference within group on VHS effectiveness or supplementary sleep subscales • A significant group by days interaction on VHS sleep disturbance with intervention group having less disrupted sleep at intervention end • Intervention group reported less perinatal worries post intervention (PAAS-P) • No difference between groups on other PAAS subscales post intervention • No difference between groups on the CES-D post intervention
**Field et al. (2013), USA **[45]	Combined form of tai chi and yoga combining balance and stretching	RCT, between group	Face-to-face, group sessions	Trained yoga instructor	One 20- minute session per week for 12 weeks	• Baseline (13–40 weeks GE) • Intervention end	92 enrolled (46 vs 46) 75 completed (37 vs 38)	82%	Prenatal ultrasound clinics affiliated with large university medical centre	CES-D-20 CES-DA CES-DS STAI-40	VSH-15	• Greater improvement in depression scores (CES-D total and subscale scores) and anxiety scores (STAI) for intervention group • Decrease in sleep disturbances for intervention group compared to an increase in sleep disturbances for control group
**Lewis et al. (2014), USA **[52]	Gradual increase in moderate to vigorous intensity physical activity with and telephone support and education sessions	RCT, between group	Individual telephone sessions	Health counsellor	11 phone sessions in 6 months (weekly in 1st month, bi-weekly in 2nd & 3rd month, in 4th, 5th & 6th month)	• Baseline (0–8 weeks PN) • Intervention end	130 enrolled (66 vs 64) 124 completed (61 vs 63)	95%	Online and traditional media, targeted emails and physician referrals	SCID-I PHQ-9 EPDS	PSQI-19	• No difference between groups for sleep quality post intervention • Intervention group fewer depressive symptoms on the PHQ-9 and EPDS but no differences by clinical interview (DSM-IV)
**Liu (Y.) et al (2016), Taiwan **[53]	Music listening of pre-recorded symphonic or classical, nature sounds, lullabies or Chinese children’s rhymes/songs CD’s	RCT, within and between group	CD, self-delivered after instruction	Not reported	30 mins listening every bedtime for 2 weeks	• Baseline (18–34 weeks GE) • Intervention end	128 enrolled (65 vs 63) 121 completed (61 vs 60)	95%	Medical centre antenatal clinic	STAI-20	PSQI-19	• Improvements in sleep quality pre-post for both groups and better sleep quality post for intervention • Improved anxiety symptoms for intervention group pre-post and fewer anxiety symptoms post for intervention
**Liu (Y.) et al. (2021), Taiwan **[54]	Exercise intervention, freeform, long-step walking exercise 20–30 min per session and three sessions per week.	Quasi experimental, non-randomised, between group	Home based, self-delivered after instruction	Research assistant	20–30 min sessions, 3 times per week for 12 weeks	• Baseline (6 weeks PN) • Mid intervention (4 weeks) • Intervention end	104 enrolled (50 vs 54) 96 completed (1 month) (47 vs 49) 88 completed (3 month) (41 vs 47)	92% (mid intervention) 85% (intervention end)	Teaching hospital	EPDS-10	PSQS-F1 PSQS-F2	• No difference between groups on depressive symptoms • No difference between groups for infant night-care-related daytime dysfunction (PSQS-F1) • Intervention group fewer physical symptoms related to sleep inefficiency (PSQS-F2) mid intervention but not post intervention
**Mindell et al. (2018), USA **[56]	Nightly two-step massage-based bedtime routine and quiet activities (e.g., cuddling and singing lullaby), lights out within 30 min after completing the full-body massage	RCT, between group	Self-delivered (after instruction)	Not reported	Nightly for 3 weeks	• Baseline (3–18 months PN) • Intervention end	Enrolled not reported 123 completed (64 vs 59)	Not reported	Independent clinical research organization	EPDS-10 BMIS-16 STAI-40	PSQI-B PSQI-SOL PSQI-Awak PSQI-DAwak PSQI-TST PSQI-19 PSQI> 5 ESS-8	• Intervention groups had reduction in number of night wakings, improved sleep quality and a significant reduction in % mothers designated as poor sleepers • No difference between groups for daytime sleepiness (ESS), bedtime, sleep latency, TST and time spent awake • No difference between groups for depressive (EDPS) or anxiety symptoms (STAI) • Intervention group had improved BMIS scores from baseline to week one and two
**Teychenne et al. (2020), Australia **[67]	Home-based physical activity including treadmill or stationary bicycle, access to smartphone app, logbook for goal setting and self-monitoring, online forum for social support	RCT, between group	Self-delivered after instruction, with online forum for feedback and support	Research assistant	12 weeks (regularity not reported)	• Baseline (3–9 months PN) • Mid intervention (4 weeks) • Mid intervention (8 weeks) • Intervention end	62 enrolled (32 vs 30) 56 completed (31 vs 25)	90%	Social media and websites and flyers at Maternal Child Health Centres	EPDS GAD-7	PSQI-3	• No difference between groups for depressive symptoms (EPDS or GAD) • No difference between groups for sleep quality
**Xue et al. (2020), China **[71]	Drinking magnolia tea	RCT, between group	Self-delivered after instruction	No personal contact	One cup per day for 3 weeks	• Baseline (newly delivered) • Intervention end • 6 weeks PN	112 enrolled (56 vs 56) 101 completed (50 vs 51)	90%	Not reported	EPDS-10	PSQS-F1 PSQS-F2	• Intervention group better physical-symptom-related sleep inefficiency immediately post intervention but not at 6 weeks postnatal • No difference between groups in sleep inefficiency related to infant night-care and daytime dysfunction • Intervention group fewer depressive symptoms immediately post intervention and at 6 weeks postnatal
**Yang et al. (2018), Taiwan **[72]	Aerobic gymnastic involving sitting and standing exercises using a DVD in the home	RCT, within and between group	DVD, self-delivered after instruction	No personal contact	15 min sessions, 3 times per week for 12 weeks	• Baseline (6 weeks PN) • Mid intervention (4 weeks) • Intervention end	140 enrolled (70 vs 70) 122 completed (60 vs 62)	87%	Medical centre postnatal clinic	EPDS-10	PSQS-14 PSQS-F1 PSQS-F2	• PSQS total score decreased in the intervention compared to control at intervention end but no significant effect of group, time or interaction • PSQS sleep inefficiency score decreased for intervention group mid and post intervention. Control group decreased (significant effect of time but not group or interaction) • Depressive symptoms decreased for the intervention group mid and post intervention. Control group decreased (significant effect of time but not group or interaction)

*BMIS-16* 16-item Brief Mood Introspection Scale, *CES-D-20* Centre for Epidemiological Studies Depression, *CES-DA* Centre for Epidemiological Studies Depression Affect subscale, *CES-DS* Centre for Epidemiological Studies Depression Somatic/Vegetative subscale, *EPDS-10* 10-item Edinburgh Postnatal Depression Scale, *ESS-8* 8-item Epworth Sleepiness Scale, *GAD-7* 7-item Generalized Anxiety Disorder Scale, *PAAS-P* Perinatal Anxieties and Attitudes Scale Pregnancy subscale, *PAAS-L* Perinatal Anxieties and Attitudes Scale Labor & Birth subscale, *PAAS-W* Perinatal Anxieties and Attitudes Scale Worries and Post Birth subscale, *PAAS-PO* Perinatal Anxieties and Attitudes Scale Pregnancy Onset subscale, *PHQ-9* 9-item Patient Health Questionnaire, *POMS-D* Profile of Mood States Depression subscale, *PSQI-3* 3-item Pittsburgh Sleep Quality Inventory, *PSQI-19* 19-item Pittsburgh Sleep Quality Inventory, *PSQI-B* Pittsburgh Sleep Quality Inventory Bedtime, *PSQI-SOL* Pittsburgh Sleep Quality Inventory Sleep Onset Latency, *PSQI-Awak* Pittsburgh Sleep Quality Inventory Number of Night Wakings, *PSQI-DAwak* Pittsburgh Sleep Quality Inventory Duration of Night Wakings, *PSQI-TST* Pittsburgh Sleep Quality Inventory Total Sleep Time, *PSQI > 5* Pittsburgh Sleep Quality Inventory (score > 5), *PSQS-14* 14-item Postpartum Sleep Quality Scale, *PSQS-F1* Postpartum Sleep Quality Scale Factor 1 (Infant night-care related daytime dysfunction), *PSQS-F2* Postpartum Sleep Quality Scale Factor 2 (Physical-symptom-related sleep inefficiency), *SCID-I* Structured Clinical Interview for DSM-IV Axis I Disorders, *STAI-20-* 20-item State Anxiety Inventory, *STAI-40* 40-item State Trait Anxiety Inventory, *VSH-15* 15-item Verran and Snyder-Halpern Sleep Scale, *VSH-D* Verran and Snyder-Halpern Sleep Scale - Disturbance subscale, *VSH-E* Verran and Snyder-Halpern Sleep Scale - Effectiveness subscale, *VSH-S* Verran and Snyder-Halpern Sleep Scale - Supplementary subscale

**Table 7:** Chronotherapeutic interventions

Author	Intervention Description	Study Design / Analytical Approach	Intervention Delivery	Intervention Facilitator	Intervention Length	Data Time Points (PN = postnatal, GE = gestational)	Number of Participants (Intervention vs Control)	Retention Rate	Recruitment Sites and Methods	Mental Health Measures	Sleep Measures	Results Summary
**Lee et al. (2013), USA **[51]	Morning bright light therapy and a 30-min discussion on principles of sleep hygiene and a sleep hygiene booklet	RCT, between group	Face-to-face individual sessions	Trained graduate research nurse	30 mins every morning for 3 weeks	• Baseline (5–10 days PN) • Intervention end	35 enrolled 30 completed (16 vs 14)	85%	Hospital neonatal intensive care unit	EPDS-10 SF36v2-M	GSDS-SQ GSDS-DF TST-NA TST-DA	• No difference between groups for sleep quality and daytime functioning or sleep duration • No difference between groups for depressive symptoms or mental health–related quality of life
**Parry et al. (2019), USA **[58]	Cross-over of one night of early-night wake therapy (EWT) and late-night wake therapy (LWT)	Single arm, pre-post	In a general clinical research centre	General clinical research centre staff	One night of either EWT or LWT separated by 1 week	• Baseline (0–34 weeks GE to 0–12 months PN) • Intervention end	50 enrolled 26 antenatal (17 healthy controls, 9 clinically depressed) 24 postnatal (8 healthy controls, 16 clinically depressed) **Study Design recast** 15 antenatal (EWT) 18 antenatal (LWT) 15 postnatal (EWT) 14 postnatal (LWT)	Not reported	Not reported	HAMD-21	Actigraphy PSG DLMO	• EWT showed greater improvement in mood in pregnant women compared to postpartum women; LWT showed greater improvement in postpartum women compared to pregnant women. • Improved mood in pregnant women after EWT was associated with less time between melatonin onset and sleep onset. Improved mood in postpartum women after LWT was associated with increased total sleep time.
**Swanson et al. (2018), USA **[66]	Morning light therapy using light therapy glasses	Single arm, pre-post	Light therapy glasses, self- delivered after instruction	No personal contact	60 mins every morning for 5 weeks	• Baseline (0–6 months PN) • Intervention end	10 enrolled 8 completed	80%	Department of Psychiatry and community advertisements	EPDS-10 SIGH-SAD	TST-D TST-A SE-D SE-A DLMO PAD	• Improvement in depressive symptoms (EPDS and SIGH-SAD) • Improvement in self-reported sleep efficiency • No change in diary or actigraphy TST, actigraphic sleep efficiency, DLMO, and PAD • Correlation between change in PAD and percent change in SIGH-SAD score (lengthening of the PAD associated with greater improvement on SIGH-SAD)

*DLMO* Dim Light Melatonin Onset, *EPDS-10* 10-item Edinburgh Postnatal Depression Scale, *EWT* Early-Night Wake Therapy, *GSDS-SQ* General Sleep Disturbance Scale Sleep Quality subscale, *GSDS-DF* General Sleep Disturbance Scale Daytime Functioning subscale, *HAMD-21* 21-item Hamilton Depression Rating Scale, *LWT* Late-Night Wake Therapy, *PAD* Phase angle difference between DLMO and midpoint of sleep per wrist actigraphy, *PSG* Polysomnography, *SF36v2-M* Medical Outcomes Short Form-36, version 2, Mental subscale, *SIGH-SAD* Structured Interview Guide for the Hamilton Depression Rating Scale, Seasonal Affective Disorders, *TST-A* Total Sleep Time (actigraphy), *TST-NA* Total Sleep Time Nocturnal (actigraphy), *TST-DA* Total Sleep Time Daytime (actigraphy), *TST-D* Total Sleep Time (diary), *SE-D* Sleep Efficiency (diary), *SE-A* Sleep Efficiency (actigraphy)

## Results

Studies were conducted in the United States (*n* = 12), Australia (*n* = 7), China (*n* = 5), Taiwan (n = 5), Canada (*n* = 3), New Zealand (*n* = 2), Iran (*n* = 1), Sweden (*n* = 1), and Turkey (*n* = 1). Fig. 2 illustrates the recent growth in this field, with 86% of studies published in the past decade (*n* = 32).

### Demographic characteristics

Twenty-eight of the eligible study samples reported a mean maternal age between 30 and 35 years, and seven studies reporting a mean maternal age between 25 and 30 years of age. Two studies did not report maternal age [53, 75]. Table 2 summarises the parity, education level, relationship status, socioeconomic position and race/ethnicity of the study samples. Based on information provided, participants in these studies were predominantly well-educated, not socio-economically disadvantaged, in stable relationships, primiparous and of White race/ethnicity.

### Eligibility criteria

Over half of the studies screened potential participants to include women experiencing moderate or severe current or previous mental health concerns and/or moderate to severe sleep disruption (*n* = 19, 51%). Approximately one third of study samples had no restrictions on participants physical, mental and sleep health status (*n* = 11, 30%). Only one study assessed potential participants to ensure those who enrolled had no problems with their physical, mental and sleep health, whereas six samples screened participants to ensure good health in one or two of these domains.

### Study design

Of the 37 interventions included in this review, 26 utilised a randomised controlled trial (RCT) study design, one study was a quasi-experimental controlled trial, and 10 studies were single arm pre-post comparisons. Across all 37 interventions, 4986 participants were enrolled, and 4422 women completed the interventions (sample size range = 10 to 802, median = 85). Sample sizes were smaller in single arm studies, (total 278 participants; range 10–85) whereas RCT study designs were generally larger (total of 4513 participants; range 26–802).

### Retention

Retention rates were calculated using data from each manuscript. The mean retention rate across all studies was 89% (range = 55 to 100%), with similar rates for the different study designs (84% single arm, 89% case-control).

### Sleep, mood and acceptability measures

Over half the studies (51%) used the Pittsburgh Sleep Quality Index (PSQI) as a sleep measure and over three-quarters of the studies (81%) used the Edinburgh Postnatal Depression Scale (EPDS) as a mental health measure. Table 3 presents the range of sleep and mental health measures used in the studies. Nine studies used objective sleep measures and only one study used clinical interview for their mental health assessment. Just over a quarter of the studies included one or more follow-up timepoints past intervention end and less than a third reported on intervention acceptability. Just over a third of interventions were online or self-delivered, with the remaining being delivered in person.

### Intervention categories

After careful review, interventions were grouped into 4 general categories to enable further description: 1) psychological, 2) educational, 3) lifestyle and 4) chronotherapeutic. Psychological interventions (*n* = 9) were those based on a treatment or therapy aimed at changing cognitions, attitudes or emotions and founded on psychological theories. Educational interventions (*n* = 15) focused on providing participants with practical information and strategies to improve or support mood or sleep (maternal or infant). Lifestyle interventions (*n* = 10) used a range of methods to intervene, including exercise, massage, listening to music, and consuming various herbal teas. The final category of interventions, chronotherapeutic (*n* = 3), included studies based around changes to the timing of the circadian system or the timing of sleep. Interventions could incorporate components from more than one category, for example, a chronotherapeutic intervention could also include some educational material but was categorised as chronotherapeutic based on the key focus of the intervention.

Figure 3 illustrates the four identified categories (inner), the focus of the intervention (middle), and the intervention timeframe (outer). Twenty-one studies were conducted in the postnatal period (57%), 14 were conducted during pregnancy (38%) and only two studies spanned both pregnancy and the postnatal period (5%). The tendency for interventions to be delivered in the postnatal period was similar across all categories of intervention, except psychological interventions, where five studies (56%) were conducted in pregnancy and four studies (44%) in the postnatal period.

Figure 4 shows the timing of recruitment and intervention phases of each study. Amongst the 14 interventions commencing in pregnancy, only one study recruited solely in the first trimester, four recruited in the first and second trimesters, seven in the second and third trimesters and three in the third trimester. The intervention phase of these studies was conducted predominantly in the second trimester; with eight conducted exclusively in the second trimester, three spanned the second and third trimesters, two studies were conducted solely in the third trimester and one commenced in the third trimester and continued into the postnatal period. Seventeen of the 22 studies conducted in the postnatal period began recruitment within 3 months of birth. Three studies began recruitment at 3 months postnatal and the remaining two studies recruited between 6 and 7 months postnatal. Across all studies, the intervention length ranged from 5 days to 6 months (mean 6.5 weeks); 26 studies (70%) lasted 6 weeks or less, eight studies (22%) lasted between 8 and 12 weeks and three studies (8%) lasted 6 months or more.

### Psychological interventions

The nine psychological interventions identified in this review centred around mindfulness (*n* = 4) [32, 50, 55, 64] and cognitive behavioural theory (CBT) (*n* = 5) [30, 31, 46, 65, 68]. Table 4 provides a summary of the interventions.

#### CBT interventions

##### Characteristics

Of the five CBT interventions, three occurred during pregnancy [30, 46, 68] and two during the postnatal period [31, 65] and ranged from five to 10 weeks in duration. Three studies used CBT for insomnia (CBTi) and included information about maternal and infant sleep [30, 65, 68], while two further studies used generic CBT and focussed on maternal mental health [31, 46]. One study did not report who facilitated the intervention or how it was delivered [31], while four studies were facilitated by a trained CBT therapist or clinical psychologist, with three conducting face-to-face individual or group sessions [30, 65, 68] and one using online delivery [46]. Improving both sleep and mood were the focus of three studies [30, 31, 68], while one study concentrated on mood as a primary outcome and sleep as a secondary outcome [65] and another study did the reverse [46]. All CBT interventions recruited women experiencing either moderate to severe depressive symptoms (*n* = 2) [31, 46], moderate or severe insomnia (*n* = 2) [30, 68] or both (*n* = 1) [65]. Three studies reported on acceptability measures: one study reported 95% of participants found the intervention important and helpful [46]; another that 77% of participants felt “satisfied” or “very satisfied” with the care provided [31]; and a further study reported 54% of participants enjoyed the group environment and 62% enjoyed the education component [68].

##### Measures and findings

In general, the CBTi interventions reported improvements in both sleep and mood, with group improvements or group differences in both sleep quality and insomnia symptoms [30, 65, 68]. One of these studies reported on sleep diary and actigraphy (a validated and objective motion-based measure of sleep) results, finding sleep efficiency was improved, however they noted mixed results for other dimensions of sleep [68]. Mental health measures were improved in all CBTi studies (depression symptoms in two studies [30, 65] and depression and anxiety symptoms in the other study [68]). The two CBT studies that utilised RCT methodology largely reported improvements in mental health measures when comparing the intervention and control groups [31, 46], however one study found improvements in two measures of mental health (MADRS and GAD) but not the third (EPDS) [46]. One CBT study found improvements in self-reported sleep quality [31], but the other did not find differences between groups for insomnia symptoms [46]. There was no follow-up past the intervention end for any of the CBT intervention studies.

#### Mindfulness interventions

##### Characteristics

All four mindfulness interventions [32, 50, 55, 64] were self-delivered or administered using apps or recordings after participants were provided with instructions. Two interventions were delivered during pregnancy [50, 64] and two during the postnatal period [32, 55] and they ranged in length from two to 8 weeks duration. Three of the four studies involved women with moderate to severe depressive symptoms [32, 50, 64]; the fourth had no eligibility criteria [55]. Improving sleep and mood concurrently were the focus of three studies [32, 50, 55], while the fourth study focussed on mood as a primary outcome and sleep as a secondary outcome [64]. Three studies reported on acceptability, with two studies noting that 67 and 69% of the sample were “very” or “extremely” satisfied [32, 50], while the other study reported that 96% of participant’s had a “somewhat” or “very positive” experience [55].

##### Measures and findings

Overall, participants in mindfulness interventions reported improvements in both sleep and mood. Two single-arm mindfulness studies reported within-group improvements immediately post intervention for both sleep quality and mental health measures (depression symptoms) [32, 50]. Another single-arm study reported a decrease in depressive symptoms and improvements in both sleep quality and duration (but not sleep efficiency or latency) [55]. Only one mindfulness intervention had follow-up time points after the intervention end and was also the only study that used an RCT [64]. This study reported between group differences on depressive and anxiety symptoms but not on sleep quality measures. It also found within group differences in depressive symptoms immediately post intervention and in late pregnancy, but not at 6 weeks postnatal, and within group differences in anxiety symptoms immediately post intervention and at both follow-up time points.

### Educational interventions

The 14 educational interventions identified in this review focussed on either maternal health (*n* = 5) [29, 57, 60, 73, 74], infant health (*n* = 5) [41, 47, 48, 59, 61] or a combination of both (*n* = 4) [49, 62, 63, 69, 70] (70 & 71 used the same sample). Table 5 provides a summary of the interventions.

#### Maternal health interventions

##### Characteristics

Five maternal health interventions [29, 57, 60, 73, 74] ranged from 4 to 24 weeks in duration. Four occurred during pregnancy and one during the postnatal period [57]. Four of the five studies used an RCT design, and all had similar session schedules: 1–2-hours in duration, weekly or fortnightly, over a time period of 4–12 weeks, the shortest consisting of four sessions over 4 weeks and the longest consisting of eight sessions over 12 weeks. The fifth study was a single arm pilot which ran 1–2-hour education sessions in the first, second and third trimesters of pregnancy (three sessions over 24 weeks) [29]. All five of these interventions utilised face-to-face delivery; three studies provided individual education sessions [29, 57, 74], one study used group education sessions [73] and one utilised both modes [60]. Four interventions were considered ‘individualised’, ‘tailored’ or ‘interactive’, catering to the sleep and health needs of the participant [29, 60, 73, 74]. The facilitators of the education sessions varied and included; a sleep scientist (*n* = 1) [29], registered nurse/midwife (*n* = 3) [57, 73, 74] and an allied health professional (*n* = 1) [60]. Two studies recruited women with either current or previous mental health symptoms [29, 74], one study included women with current mental health symptoms as well as an obstetric complication [73], and two studies had no eligibility criteria [57, 60]. The content of the interventions ranged widely. One study focussed on improving sleep [29], two centred on obstetric issues and mental health concerns [73, 74] and two had a broader lifestyle and health focus (e.g., nutrition, exercise, sleep, stress) [57, 60].

##### Measures and findings

Improving both sleep and mood were primary outcomes for four studies, and one study concentrated on reducing weight gain as a primary outcome and sleep and mood as secondary outcomes [60]. Significant results were found for all primary outcomes, but no change was seen for any secondary measures in these studies. The three studies measuring sleep quality and the two studies reporting on insomnia symptoms found improvements within or between groups [29, 57, 60], particularly for sleep quality, but not across all follow-up time points [29] or all dimensions of sleep [29, 57]. Two of the three studies investigating sleep duration found that intervention groups slept longer [73, 74] however the one study examining daytime sleepiness found no difference between intervention and control groups [29]. Depressive symptoms improved in three out of the six studies [29, 73, 74], but not at follow-up for one [29]. One study that examined overall psychological health found that the intervention group remained stable while the control group deteriorated [57]. The two studies examining anxiety symptoms found no difference between groups [29, 60].

Only one study reported on acceptability measures, with 86% of participants reporting the intervention was ‘definitely’ a positive experience and 93% reporting they would ‘definitely’ recommend the study to others [29]. This was also the only study to have follow-up measures past the conclusion of the intervention.

#### Infant sleep interventions

##### Characteristics

Five studies were classified as infant sleep interventions [41, 47, 48, 59, 61], ranging from 2 to 26 weeks in duration. Four of the interventions were similar, in that they were conducted solely during the postnatal period. Only one intervention spanned late pregnancy through until 4 months postnatal [47]. All studies had relatively large cohorts with between 72 and 802 enrolments and high retention rates (> 82%). Only one study had health requirements for eligibility (good mental, physical and sleep health) [59], while the remaining studies had no mental, physical and sleep health criteria.

Four of the five studies were similar in their aims and delivery style: all aimed to provide information about infant sleep and/or establish infant sleep management plans via an initial face-to-face individual session, with subsequent weekly or fortnightly follow-up sessions (either face-to-face or telephone) over a 2–8-week period [41, 48, 59, 61]. These four interventions were delivered by health professionals (midwife, paediatrician or maternal health nurse). All but one of these interventions (which did not provide information) [59], reported that the sessions were either ‘individualised’ or ‘tailored’ to the mother’s (and in one case, mother and father’s [61]) concerns about their infant’s sleep.

The fifth study varied from those above, and was a longer 6-month RCT intervention with four arms covering a more holistic range of material, primarily infant sleep, with additional support on breastfeeding, nutrition and exercise (supplementary to normal infant care from an infant health nurse) [47]. This intervention combined group and individual sessions, included partners and was facilitated by a researcher with infant sleep training and a lactation consultant.

##### Measures and findings

Two of the five infant sleep education interventions identified maternal sleep and mood as primary outcomes [47, 59], while another study also had maternal mood as a primary outcome, with maternal sleep as a secondary outcome [41]. The two remaining studies assessed infant sleep (*n* = 2) as primary outcomes with maternal mood and sleep as secondary outcomes [48, 61]. Three of the five studies had follow-up periods past the intervention end date [41, 47, 48]. Three of the five studies reported improvements in maternal sleep quality (either pre-post or between groups) immediately post intervention [41, 59, 61] and one study saw improvements at the 2-month follow-up but not immediately [48]. Maternal sleep duration results were mixed, with two studies reporting improvements (one immediately [41] and one at follow-up [48]), while two studies saw no difference or improvement [47, 61]. Four of the five studies saw improvements in maternal mood immediately post intervention [41, 48, 59, 61], however, the two studies that included follow-up at 2-months post intervention had conflicting findings on the longevity of these improvements [41, 48].

Four of the five studies measured acceptability. Helpfulness was rated in two studies; one intervention was rated by 75% of women as helpful [61] and another had ratings of 73–100% for the helpfulness of individual strategies [59] (e.g., ‘putting infant in bed, awake but drowsy’). The remaining two studies rated satisfaction and usefulness on visual analogue scales (8.2 and 8.4 (out of 9) [41], the other 7.7 and 7.3 (out of 10) respectively [48]).

#### Combined maternal and infant sleep interventions

##### Characteristics

Although there were four interventions that encompassed both maternal and infant sleep, two were very similar, being a pilot [62] and RCT [63] of the same TIPS (Tips for Infant and Parents Sleep) program. This intervention was similar to the infant interventions describe above, in that a nurse guided an initial face-to-face individual session, with subsequent weekly follow-up telephone calls over a 4- to 5-week period that were ‘individualised’ to the mother’s concerns. Another maternal/infant intervention was a 5-day intensive residential program, with a range of medical clinicians providing multidisciplinary assistance and support to mother and baby (two manuscripts providing different data on the same intervention) [69, 70]. The last maternal/infant sleep intervention was an RCT study, providing sleep information and strategies to women and their partners over two 1.5 hr. face-to-face group sessions in late pregnancy [49]. It had three follow-up data time points in the postnatal period and was facilitated by a sleep psychologist.

##### Measures and findings

While the TIPS pilot study found sleep duration and self-reported sleep problems were significantly different between groups (but not sleep quality, 24 hr. TST, and sleep disruptions), the full TIPS RCT did not. Mood related measures did not differ in either the pilot or full RCT. Acceptability and usability measures were well described with only one woman in the pilot [62] and eight women in the RCT indicating that would not re-participate [63]. The residential program intervention found improvements to mood outcomes and most sleep related measures (except duration) but did not report on follow-up data or program acceptability [69, 70]. The joint mother and partner RCT study focussed on sleep as a primary outcome and found differences in sleep quality and insomnia symptoms at some but not all time points [49]. No differences were seen for daytime sleepiness. Depression and anxiety symptoms were secondary outcomes, and no differences were seen on either measure. No acceptability measures were reported.

### Lifestyle interventions

Eleven studies were classified as lifestyle interventions, including; drinking herbal tea (*n* = 3) [42, 43, 71], listening to music (*n* = 1) [53], massage (*n* = 2) [44, 56] and incorporating exercise/movement (*n* = 5) [45, 52, 54, 67, 72]. Table 6 provides a summary of the interventions.

#### Herbal tea interventions

##### Characteristics

The three herbal tea interventions were largely similar in design; all were RCTs requiring women to drink one cup of tea a day for 2–3 weeks and all were aimed at women experiencing poor sleep health in the early postnatal period [42, 43, 71].

##### Measures and findings

All three studies used the PSQS to assess sleep and the EPDS to evaluate mood (both primary measures) with measures completed immediately post intervention and an additional follow-up timepoint 2–3 weeks past the intervention end. Both the chamomile and magnolia tea interventions found the intervention group to have improved physical sleep related symptoms immediately post intervention, though not at follow-up, and fewer depressive symptoms at both post intervention time points compared to controls [42, 71]. The lavender tea intervention did not find a between group difference in sleep and although depressive symptoms were better immediately post intervention, this difference was also apparent at baseline and did not extend through to the follow-up timepoint [43]. Study samples for the three studies ranged between 80 and 112 participants and retention rates were above 90%, however none of the studies reported on intervention acceptability.

#### Music interventions

##### Characteristics

One RCT intervention explored listening to music as a strategy for improving sleep and mood [53]. This intervention was aimed at women in their second trimester experiencing poor sleep. Like the tea drinking interventions, music was self-guided over 2 weeks, with women required to listen to one of five pre-recorded CDs for a minimum of 30-minutes at bedtime.

##### Measures and findings

Both the intervention and control groups reported better sleep quality, with the intervention group having greater improvement compared to controls. Within and between group differences were found for anxiety symptoms at intervention end. Participant retention was 95% and there were no follow-up time points past intervention end or acceptability information.

#### Massage interventions

##### Characteristics

Two interventions utilised massage, with very different study designs. One intervention investigated the effect of ten 20-minute massages over 5 weeks for women in their second trimester, delivered by trained massage therapists [44]. The second intervention was conducted in the second half of the first postnatal year, with 123 mothers massaging their infants nightly over 2 weeks [56]. The only physical, mental or sleep health eligibility criteria was for the infant massage intervention which required mothers to be physically healthy.

##### Measures and findings

Even though the recipients of the massage were different, maternal sleep and mood were primary measures in both studies. Within and/or between group sleep and mood results were varied. The maternal massage study found sleep disturbance improved for the intervention group from baseline to intervention end but there was no difference for other sleep metrics [44]. This study found improvements in immediate (beginning to end of massage) depressive and anxiety symptoms and longer-term improvements (baseline to intervention end) on the PAAS Pregnancy subscale, but not on the other PAAS subscales or on the CES-D. The infant massage study found mothers in the intervention group had greater improvement in sleep quality, fewer night wakings and fewer mothers were designated as poor sleepers post intervention [56]. However, there was no difference between groups for daytime sleepiness symptoms, bedtime, sleep latency, sleep duration or the time spent awake at night. Similarly, there were mixed findings for mood, with no difference between groups for depressive or anxiety symptoms, but a significant improvement in mood from baseline to weeks one and two. This study also reported that between 83% (week one) and 91% (weeks two) of mothers were ‘somewhat satisfied’ or ‘very satisfied’ with the routine and 69% of mothers were ‘very likely’ to continue the recommended routine in the future.

#### Exercise/movement interventions

##### Characteristics

Five studies were movement or exercise-based interventions [45, 52, 54, 67, 72]. Four studies used RCT and one study employed a quasi-experimental [54] study design, with relatively large cohorts (62–140 enrolments) and high retention rates (> 82%). The sole intervention occurring in pregnancy was a weekly 20-minute tai chi/yoga group session, delivered face-to-face by a qualified yoga instructor over 3 months [45]. The four postnatal interventions were similar in design to each other, in that they involved self-delivered (after instruction) moderate to high intensity aerobic exercises involving either walking [54], gymnastic exercises [72] or home exercise equipment (stationary bicycle or treadmill) [52, 67]. Three of the four aerobic interventions lasted 3 months [54, 67, 72] with only one of these studies providing contact and support to participants throughout, via access to an online forum [67]. The fourth aerobic intervention lasted 6 months and was the only study in this category that provided 11 individualised support phone calls and also covered additional health information (stress reduction, nutrition and sleep) [52]. Three studies recruited women with either current or previous poor mental health [45, 52, 67] and one study recruited women with poor sleep [54]. The remaining study had no mental, physical and sleep health criteria [72].

##### Measures and findings

Three exercise interventions identified maternal sleep and mood as primary outcomes [45, 54, 72], while another study also had maternal mood as a primary outcome, with maternal sleep as a secondary outcome [52]. The remaining study assessed feasibility as its primary outcomes with maternal mood and sleep as secondary outcomes [67]. While three of the five studies had mid-intervention data collection time points, none of the five studies had follow-up periods past the intervention end date [54, 67, 72].

Only one study (the face-to-face yoga/tai chi intervention) observed between group differences on both sleep and mood [45]. The 6-month study, with support phone calls, found mixed findings for mood measures, with participants reporting fewer depressive symptoms post intervention on the EPDS and PHQ, but not when assessed using clinical interview (DSM-IV) [52]. There were also no changes in sleep in this study. The three remaining self-delivered studies had either no improvements in mood or sleep measures [67], reported significant differences in both the intervention and control groups [72] or reported a group difference on one sleep measure (fewer physical symptoms related to sleep inefficiency) mid intervention but not at intervention end [54].

Two of the five studies measured acceptability, with one study reporting specific health and wellbeing benefits (i.e., reducing muscular pain) for between 36 and 50% of participants [72]. The other study had a key focus on feasibility and acceptability and reported comprehensively on these topics [67] and whilst exact percentages were not reported, it was stated that ‘almost all women’ liked the convenience, accessibility and flexibility of the program and that ‘a majority’ of women suggested it had a positive effect on exercise engagement.

### Chronotherapeutic interventions

Two studies used bright light therapy (BLT) [51, 66] and one study trialled sleep restriction in either the first or second half of the night [58]. Table 7 provides a summary of the interventions.

#### Bright light therapy interventions

##### Characteristics, measures and finding

The two BLT studies required women, who were early in the postnatal period, to wear light therapy glasses (visors) daily for 30–60 minutes within the first hour of waking. One of the BLT interventions also included a brief discussion of sleep hygiene principles prior to using the glasses [51]. This 2-week intervention, that utilised an RCT design, found no difference for either sleep or mood post intervention. This small study (*n* = 10) found improvements in depressive symptoms and sleep efficiency, but no improvement in other aspects of sleep. It also found a significant correlation between the phase angle difference (PAD) of melatonin and sleep onset and percent change in SIGH-SAD score (lengthening of the PAD was associated with greater improvement on SIGH-SAD). One study discussed acceptability, reporting high adherence rates to the treatment protocol and that the device was well tolerated by participants [66].

#### Early-night and late-night wake therapy intervention

##### Characteristics, measures and finding

Recruitment for this study spanned the entire perinatal period except for the 6 weeks prior to birth [58]. It involved a single-arm cross-over trial of one night of early-night wake therapy (EWT, sleep between 3:00–7:00 am) versus late-night wake therapy (LWT, sleep between 9:00 pm–01:00 am) in physically healthy women. EWT showed greater improvement in mood in depressed pregnant women compared to postnatal women; LWT showed greater improvement in mood for depressed postpartum women compared to pregnant women. Results also showed that improved mood in pregnant women after EWT was associated with smaller PAD i.e., less time between melatonin onset and sleep onset. Improved mood in postpartum women after LWT was associated with increased total sleep time. This study did not report on intervention acceptability.

## Discussion

This scoping review identified and summarised the range and nature of 37 perinatal interventions that aimed to influence sleep and mood outcomes. The studies were grouped into either psychological, educational, lifestyle or chronotherapeutic categories depending on the intervention’s key focus and ranged from those grounded in sleep and circadian science to those based on complementary and alternative therapies. Our literature search showed that this field is rapidly growing, indicated by the number of studies published in the last 5 years, most frequently in developed Western countries (USA, Australia, New Zealand and Canada).

Most interventions started within 3 months of birth and were delivered across a relatively short period of time. The short delivery timeframe is likely to be advantageous from a participant burden perspective and assist with engagement and retention. However, retention rates did not appear to be lower for longer interventions. On the contrary, most studies that provided data showed high retention rates, despite occurring during a challenging and busy life stage. These high retention rates suggest that women are interested in, and are open to, support during the perinatal period, particularly in relation to sleep and mental health.

For some women, mental health concerns that commence in pregnancy are chronic and remain years later [29]. However, there is reliable evidence that intervening in sleep and mental health early in pregnancy [76], may prevent the onset of difficulties later in pregnancy and postnatally. However, only four of the 37 interventions in the review recruited women in their first trimester of pregnancy highlighting a gap in early and preventive perinatal interventions. Additionally, only two interventions spanned both pregnancy and the postnatal period and, as far as we are aware, there are no perinatal health interventions with a sleep or mental health focus that cover pre-conception to pregnancy even though women have voiced a strong desire for information at this time [77]. Future interventions may better support women by spanning a longer period and broadening their focus as factors that influence both sleep health and mental health change across this timeframe. For example, shifting from solely focusing on maternal sleep during pregnancy to also including information and strategies to support infant sleep.

Over half of the studies were conducted with women with existing sleep or mental health problems, and the sleep health and mental health of study samples were described for many of the studies, which is expected given the focus of the interventions. In contrast, there was often limited information provided on other demographic characteristics of the study samples, particularly the socioeconomic position and race/ethnicity of women. Only two interventions were specifically designed for, or trialled with, women from minority or disadvantaged groups [45, 51] (i.e., women from indigenous or minority ethnic groups, women experiencing socio-economic disadvantage, women with low education/literacy levels or teenage mothers). Interventions that meet the needs and priorities of these women are vital given women who experience disadvantage are disproportionately affected by poor mental and sleep health [78–80].

None of the studies reviewed explicitly stated that the interventions were designed in collaboration with women, although it is possible that this did occur but was not acknowledged. To ensure the content and focus of the information and intervention is appropriate, culturally based information is incorporated [81, 82], and suitable methods and modes of delivery are considered, interventions need to be co-designed with perinatal women, their family members, and relevant clinical, health and community stakeholders. In Aotearoa New Zealand, for example, perinatal sleep and mood interventions must be developed by or in collaboration with Māori and Pacific women, family and whānau, and Māori and Pacific health providers. This approach has been shown to be effective in the design and development of other public health interventions in minority populations [83, 84].

It is also important to note that all studies in this review were person-centric and focused on changing a woman’s thoughts or behaviour (or infant’s behaviour). Intervention at an individual level has an important role, but the structural and social determinants of mental health and sleep health, such as racism, socioeconomic deprivation, poor housing, limited education, violence, and chronic life stress are also critically important in the perinatal period [85] and must be addressed through policy and action by government and associated agencies, and community engagement [86]. Thus, alongside the development and application of perinatal interventions there must be a broader range of work to reduce the social drivers of sleep health and mental health inequities for women.

Women with existing sleep or mental health problems may also experience multiple comorbid issues including, for example, other health conditions, alcohol and substance abuse, and dietary concerns. Furthermore, in many countries, Aotearoa New Zealand included, health services that can provide support for sleep or mental health concerns and deliver interventions are very limited [87] and are often only able to engage with women who are experiencing the most severe difficulties. Findings from the review indicate that interventions can be delivered using a range of methods from online delivery with no or minimal personal contact through to live-in residential programmes. Given the restrictions created through the COVID-19 pandemic, the continued use of online information and virtual visits in delivering such interventions is important to explore, but access and effectiveness for all women must also be considered. While the reviewed interventions hold promise, barriers to accessibility and help-seeking must be taken into account and interventions may need to incorporate self-recognition of issues, encouragement to seek help and pathways to care.

Although there was some overlap between the methods used and focus of interventions, such as education in conjunction with psychological therapy, there is potential for greater integration. Together these findings suggest the following: there is space for prevention therapies to work alongside intervention models of care; that sleep and mood interventions could be integrated into routine perinatal care and support for other issues that perinatal women may be facing; and that interventions should be provided within a stepped care model and span a broader range of methods depending on the woman’s health concerns (mild vs severe symptoms, comorbid vs singular issues) or ability to participate in different formats (online vs in person delivery). For example, empirical, high quality, easily accessible information about sleep and mental health could be provided to all women via websites, apps or written material that also address a range of perinatal topics. Women that begin or continue to experience difficulties with sleep and/or their mental health need to be able to access further support in a timely manner. This might involve women being able to enrol or engage with an intervention directly and/or assessment by an informed health care provider and referral to appropriate services. Depending on the issues women face and the degree of severity, intervention options may need to be both specific (i.e., a course of bright light therapy) or broad (i.e., further education in conjunction with CBTi and admission to residential care). Proximity to care, availability of technology and therapy preference are also important options to consider when women are choosing perinatal care that is right for them.

A clear finding from the review was the lack of follow-up timepoints to determine if any improvements in either sleep or mental health persisted over longer periods of time. Those that did collect follow up data show mixed findings (long term improvement in five studies, short term but no long-term improvement in six studies, no short term or long-term improvement in eight studies, and no short-term improvement but long-term change in two studies). A recently published study found that CBTi delivered in a community sample over multiple time points during pregnancy and postpartum was associated with improved insomnia severity and sleep disturbance in late pregnancy and at 24 months postpartum, but not at 12 months postpartum [88], suggesting that within the immediate postpartum timeframe making measurable changes to sleep may be difficult to achieve but that there are long term benefits to supporting sleep at this time.

Across all interventions, a majority reported improved sleep and mood and the primary study outcomes tended to be significantly changed by the intervention. Although this sounds encouraging, it also highlights a possible bias in the publishing of studies with positive effects for sleep and mood and in the participating women who enrol in studies focused on these health outcomes.

As is the norm for scoping reviews, it was not the purpose of the present review to assess the quality or efficacy of studies or to comment on whether one category or type of intervention may be more or less effective. However, there is certainly a need for future research that evaluates the efficacy, acceptability and cost effectiveness of different types of interventions. In doing so, the clinical implications of results must also be considered. This may prove challenging, as the present review found limited and varied use of clinically significant outcome measures, such as clinically validated thresholds for sleep and mental health scales. Consideration must also be given to the rigour of measures employed in assessing intervention efficacy. Only one study in the present review utilised structured diagnostic interviews to determine the presence of mental health disorders and only nine studies employed objective measures of sleep, with the large majority relying on self-report measures. Furthermore, only 12 studies in this review reported on acceptability. It is recommended that studies seek acceptability feedback from participants to allow future perinatal interventions to be tailored and further refined to women’s needs.

Limitations of the review include limiting the review to studies published only in the English language, after 1975. The review used online databases only, but these are considered to contain most of the peer-reviewed health-related research. Accordingly, it is possible that some studies were not identified using the search strategies outlined in this paper. Exclusion of studies that did not fit the methodological criteria or were outside the designated perinatal timeframe was undertaken by one author, but the remaining 184 full text articles were considered independently for inclusion by two authors. Although important, interventions exploring infant sleep and infant health outcomes were outside the scope of this research and not included as part of this review.

## Conclusions

In summary, there is a rapidly growing body of literature on sleep and mood focused interventions during the perinatal period which indicates the importance of this field. The high prevalence of sleep and mood disturbances in the perinatal period can have severe and extended repercussions for mothers, children, families and communities and perinatal sleep complaints and mental health problems remain widely under-recognised, under-researched and under-treated. Due to the strong bi-directional relationship between sleep and mood, treating or preventing issues in one area has great potential to treat or prevent concerns in the other. Sleep is also a less stigmatised pathway through which mental health concerns can be addressed. We recommend that future interventions consider supporting perinatal women over an extended period of time using a stepped-care model, such that basic sleep and mood information is readily available to all women as part of routine perinatal care which could prevent problems occurring or issues escalating, and that as required, women can access an integrated range of therapies that are specific to their needs. The development of these perinatal interventions must involve and consider the needs of women from minority groups or women experiencing disadvantage who are disproportionately affected by poor sleep health and poor mental health in the perinatal period.

## Data Availability

The data that support the findings of this study are available from author, T.L. Signal.
